# Building better barriers: how nutrition and undernutrition impact pediatric intestinal health

**DOI:** 10.3389/fimmu.2023.1192936

**Published:** 2023-07-21

**Authors:** Sarah F. Andres, Yang Zhang, Madeline Kuhn, Brian Scottoline

**Affiliations:** ^1^ Division of Pediatric Gastroenterology, Department of Pediatrics, Oregon Health and Science University, Portland, OR, United States; ^2^ Division of Neonatology, Department of Pediatrics, Oregon Health and Science University, Portland, OR, United States

**Keywords:** human milk, undernutrition, immune system development, intestinal epithelium, digestion

## Abstract

Chronic undernutrition is a major cause of death for children under five, leaving survivors at risk for adverse long-term consequences. This review focuses on the role of nutrients in normal intestinal development and function, from the intestinal epithelium, to the closely-associated mucosal immune system and intestinal microbiota. We examine what is known about the impacts of undernutrition on intestinal physiology, with focus again on the same systems. We provide a discussion of existing animal models of undernutrition, and review the evidence demonstrating that correcting undernutrition alone does not fully ameliorate effects on intestinal function, the microbiome, or growth. We review efforts to treat undernutrition that incorporate data indicating that improved recovery is possible with interventions focused not only on delivery of sufficient energy, macronutrients, and micronutrients, but also on efforts to correct the abnormal intestinal microbiome that is a consequence of undernutrition. Understanding of the role of the intestinal microbiome in the undernourished state and correction of the phenotype is both complex and a subject that holds great potential to improve recovery. We conclude with critical unanswered questions in the field, including the need for greater mechanistic research, improved models for the impacts of undernourishment, and new interventions that incorporate recent research gains. This review highlights the importance of understanding the mechanistic effects of undernutrition on the intestinal ecosystem to better treat and improve long-term outcomes for survivors.

## Introduction

Malnutrition encompasses conditions of both undernutrition and overnutrition in terms of energy intake and includes disorders of inadequate vitamins and minerals (1). This review will focus on malnutrition in the form of undernutrition.

**Figure 1:**
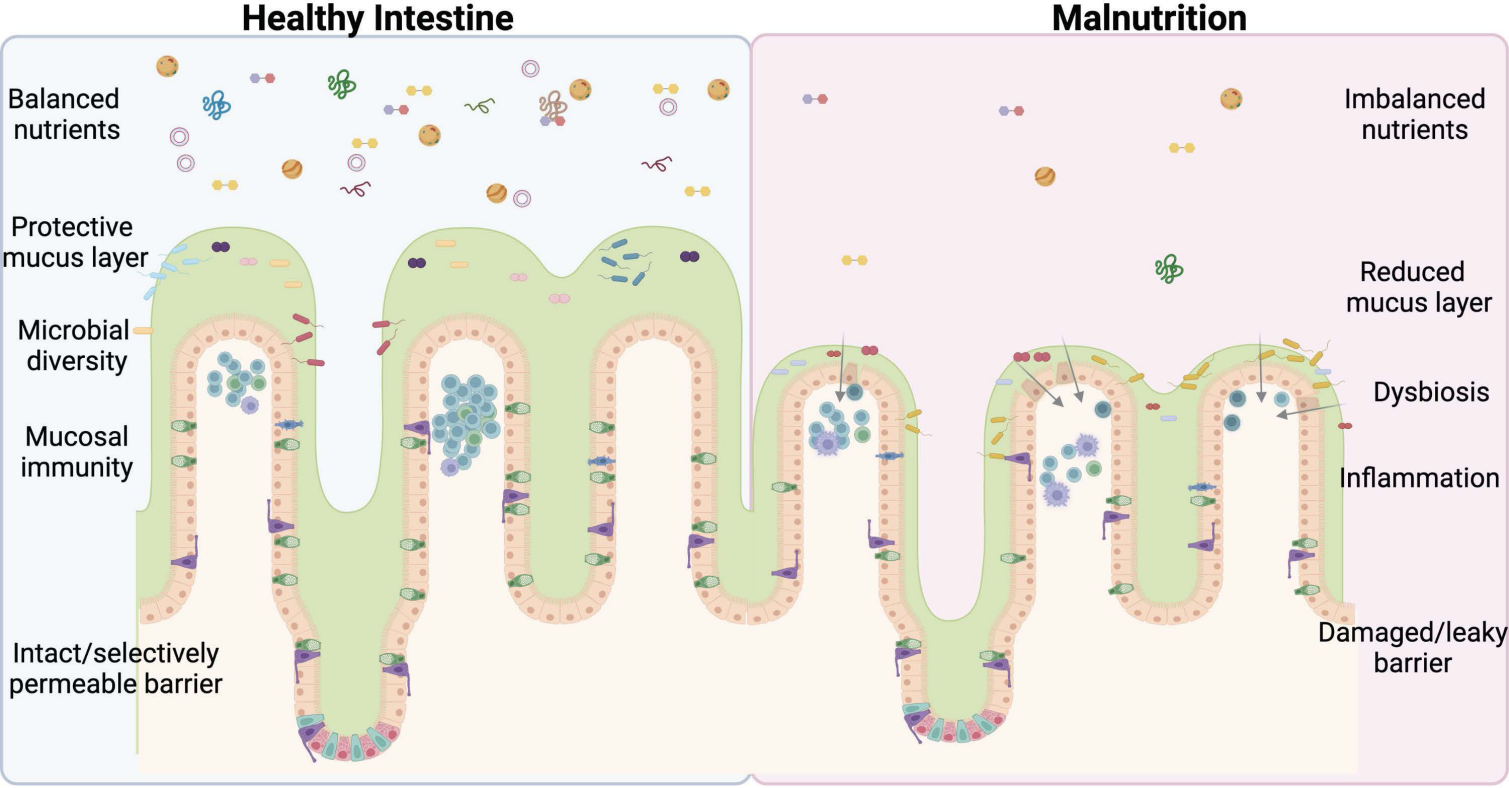
Undernutrition negatively impacts every facet of intestinal health. An imbalance of lumen nutrients is detrimental to the mammalian intestine. The villi atrophy in the absence of balanced nutrients, including Goblet cell loss and reductions in the protective mucus layer. Less mucus and varied nutrients allow for bacterial retention, dysbiosis, and infection, exacerbated by deficiencies in the mucosal immune system and ultimately culminating in barrier damage and leakage of lumen contents into the submucosal space. This perpetuates a vicious cycle further promoting inflammation and permitting bacterial invasion. The ideal treatment and cure for undernutrition will address each of these facets of intestinal health. Figure composed using BioRender.

Nearly half (45%) of all deaths in children under 5 are linked to undernutrition (1). Severe acute malnutrition (SAM) consists of both nutritional edema (Kwashiorkor) and Marasmus (severe wasting) (2). Malnutrition significantly alters the structure and function of the intestine and is a major contributor to illness and death in children worldwide (1). Stunting is one long-term effect of malnutrition that is not significantly reduced by providing supplemental food (3). This demonstrates that simply restoring balance to the diet is insufficient to correct long-term pathological changes and points to underlying changes in the intestinal physiology, which impact nutrient digestion and absorption that must be remedied before increased nutrient intake can functionally impact growth. There are several groups, such as Bandsma et al. (2, 4–7) looking at the physiological impact of undernutrition on the intestine, however, more studies are urgently needed to mitigate and potentially reverse the damage caused by this deadly condition. This review discusses normal intestinal development and digestive function, including the closely associated immune and microbiome compartments under normal nutrient conditions and what is known about the effects of undernutrition on GI tract function (**Figure 1**). We then aim to highlight areas that require further study and critical unanswered questions in the field with the ultimate goal of improving the survival and lives of millions of children worldwide.

## Intestinal development

The intestinal epithelium refers to the single layer of intestinal epithelial cells (IEC) that lines the luminal surface of the intestine. These cells form the most significant barrier within the human body and exist at the interface between ingested nutrients and the body cavity. The IEC barrier coordinates nutrient digestion and absorption, bacterial interactions, immune cell modulation, cell proliferation, and cell death (8). Regulation of these processes and maintenance of the barrier is critical to intestinal and organismal health.

The human intestine develops from the hindgut endoderm during weeks 3-7 of human embryonic development. The hollow tube that will become the intestinal tract elongates, and the luminal surface area increases through the eventual folding and evagination of the epithelial cell layer to form crypt evaginations that house the intestinal stem cells (ISC) and finger-like projections called villi. Villi increase the absorptive surface of the intestine by 6.5-fold when compared to a surface without villi to achieve a surface area of ~ 30m^2^ in an adult human (9). Much is still unknown about how the intestine forms and the gene expression programs that govern epithelial remodeling. For a more detailed discussion, we refer readers to this detailed review (10). The fully developed intestinal epithelium is composed of multiple specialized cell types that work in concert to perform the digestive, absorptive, secretory, and barrier functions of the intestinal epithelium (11).

The human intestinal epithelium is the most dynamic tissue in the human body, turning over every 3-5 days (12). IEC renewal is driven by a small pool of ISCs (13, 14), which continuously divide to replenish the stem cell pool and generate transit-amplifying progenitor cells to maintain the entire intestinal epithelium. Absorptive enterocytes comprise >80% of IECs and arise from highly proliferative transit-amplifying progenitor cells (13, 15), while the secretory cell types arise from a common secretory progenitor (11). Goblet cells migrate onto the villi where they secrete mucus, which serves as an additional barrier and protects the IEC from bacteria (16). Enteroendocrine cells are also largely present on the villi, where they respond to nutrients within the lumen and secrete hormones and growth factors that contribute to intestine growth (17–19). Tuft cells are rare cells comprising <1% of all IECs. These sensory cells coordinate signaling from luminal microbes, the host immune system, the enteric nervous system, and the intestinal barrier itself (20). Paneth cells migrate down to the base of the crypt; intercalated between the ISC, Paneth cells secrete ISC niche factors and antimicrobial peptides to regulate the intestinal microbiota (21, 22). Lastly, M cells are an epithelial component of Peyer’s patches that sample luminal bacteria and antigens and transport these factors to tissue macrophages and lymphocytes below (11). Peyer’s patch development and function will be discussed in more detail in the intestinal and systemic immunity section.

These epithelial cell types are joined together by tight junctions to form a selectively permeable barrier between the body and the complex luminal environment of digestive contents, chemicals, and bacteria. Maintaining an intact epithelial barrier is key to proper intestinal function, appropriate nutrient absorption, regulation of bacterial diversity, and balanced immune cell activation.

At birth, the intestinal barrier is somewhat permeable (23–26) allowing for immune system priming with select intestinal bacteria, as well as transport of large intact proteins, such as immunoglobulins from human milk into the body (27). Within days the barrier rapidly closes, typically as a result of human milk feeding and interactions with the developing microbiota (26), to prevent unwanted immune activation, bacterial translocation, or transit of undigested nutrients (23–25, 27). This barrier consists of epithelial cells joined together by tight junctions, adherens junctions, and desmosomes, which form a strong seal between adjacent cells (28). Tight junctions are composed of lipid and protein components in which variations can alter barrier permeability (29). They allow for the selective restriction of intestinal bacteria but the regulated flux of ions and molecules through the epithelial layer. Tight junctions are stabilized by adherens junctions and desmosomes (30, 31). Selective movement of nutrients and ions is mediated through the paracellular pore (32–34) and leak (35, 36) pathways, which mediate the passage of molecules based on their size and or charge as in the pore pathway. Inflammation, infection, or damage are examples of barrier breach, opening the unrestricted pathway and allowing for the passage of ions, nutrients, and bacteria across the barrier (37, 38). Rapid sealing of the barrier and restitution of the lost epithelium requires cellular proliferation and expansion. For a more detailed discussion on intestinal barrier function, we refer readers to this review (28).

## The effect of nutrition on the intestinal epithelium

Intestinal barrier maintenance is a highly energetic process closely coupled to the presence or absence of nutrients. This is exemplified by snakes whose intestine rapidly rebuilds upon feeding and atrophies after digestion is complete to conserve energy that would be consumed by continuous epithelial upkeep (39). Similar less dramatic adaptations occur within the mammalian gut in response to dietary changes (40–44), primarily driven by nutrient responsive ISC.

Modulation of calorie intake expands and contracts ISC populations. In instances of calorie restriction, where calorie consumption is reduced but dietary balance is maintained, ISC and other niche cell types expand and differentiation is decreased (40–43). This process is driven through mTORC1 and SIRT1 activation, both of which are core regulators of cellular metabolism (40, 42) and PPAR-gamma downstream of fatty acid oxidation (43). ISCs expand in response to fasting ketone bodies and rely on oxidative phosphorylation and lactate produced by glycolytic Paneth cells for their metabolism (45, 46). This may be a protective mechanism to ensure the continued survival of ISC when nutrients are scarce. Similar to a fasting snake, this pool of ISCs sits ready to differentiate into functional daughter cells in response to nutrient intake (40). Differentiation into secretory cell types is driven by Notch gene expression and stimulated by dietary fat and glucose consumption (46). ISC differentiation is also linked to nutrient metabolism as reactive oxygen species generated by ISCs contribute to the differentiation of daughter progenitors (45). These changes are in contrast to stem expansion and uncoupling from Paneth cell niche signals that occurs with high fat diet feeding (44, 47, 48) and can lead to cancer (44, 49).

ISC and the epithelium at large are also responsive to specific dietary nutrients. For example, the human and murine intestines respond to dietary vitamin D by enhancing stem cell proliferation and strengthening the intestinal barrier (50, 51). Interestingly, dietary activation of the aryl hydrocarbon receptor (AHR) via feeding of compounds present in green, leafy vegetables can limit proliferative signals and promote functional differentiation within the intestinal epithelium (52). In animal models, loss of AHR results in stem cell expansion, reduced differentiation, and compromised barrier function (52).

In animal studies of calorie restriction, reduced calorie consumption expanded the stem cell pool and increased the regenerative capacity of the intestine in response to discrete insults (40, 43, 46), suggesting that reduced calorie consumption may be beneficial to promote intestinal health with aging or in preparation of targeted damage, such as radiation treatment. However, these benefits of priming the intestine for regeneration in the face of injury are not seen in the malnourished state; in fact, undernutrition increases risk of disease and intestinal barrier breach in the face of infection or insult. One could speculate that imbalances in nutrient-coupled proliferation/differentiation signaling pathways (such as AHR) contribute to barrier disruption and inflammation in malnutrition, either directly through IEC or indirectly through the immune system (53–57).

## Early infant nutrition

Human milk is the ideal nutrition for infants, with numerous benefits, including reducing all-cause mortality in the first year of life and, compared to bovine-based formula, improved neurodevelopment (58–61). Data suggests the benefits of human milk are dose-dependent (58). Thus, the provision of optimal amounts and composition of human milk as nutrition during early infant life is critical to the current and lifelong health of the developing child. Undernutrition beginning early in the infant years would be expected to decrease the benefits conferred by milk. Surprisingly, maternal milk production volume is protected in the setting of maternal undernutrition; only in severe malnourishment is milk volume or macronutrient content impaired (62–64). Although the lactating mother will scavenge energy and macronutrients from body stores to preserve milk production, micronutrient content can be impacted (65). Despite the preservation of milk volume production, impaired infant growth has been observed in infants who breastfeed from mothers with malnourishment **(** 66 **)**. While some of this may be due to maternal micronutrient deficiencies transmitted to the infant, there may be a contribution from increased infant inflammation (64).

Human milk is a complex fluid with thousands of proteins, lipids, oligosaccharides, microRNAs (miRNAs), maternal cells, and metabolites, which have been selected for millennia to provide optimal infant nutrition and development (67). In addition to the provision of energy and nutrients, milk is a system of bioactive proteins, lipids, sugars, miRNAs, and metabolites that assist in infant development, with examples being intestinal, immune, and central nervous system development (67, 68) and development of the intestinal microbiome (69–72). It remains to be defined if severe maternal undernourishment impacts the bioactive components of milk in a manner that might adversely affect early infant development and growth.

## Early infant digestion and milk

There are several ways that undernutrition may alter human milk digestion, particularly with regard to human milk proteins. Human milk proteins are often divided into two classes, caseins and wheys. Wheys are highly proteolyzed during digestion and caseins are less degraded. Caseins include proteins with bioactivities in their intact forms, such as lactoferrin, immunoglobulins, and growth factors (73). It is unknown whether the human milk proteome is affected by maternal undernutrition or malnutrition. The proportion of bioactives in human milk may differ in abundance or ability to survive digestion in undernutrition than in the well-nourished state. There is evidence that human milk proteins may encode cryptic bioactive peptides with activities important for infant health (67). These include enterocyte and monocyte immune modulation (74, 75) and modulation of bacterial growth and survival (76). How undernutrition may alter the production of these milk protein digestion products is unknown. Moreover, how undernutrition could alter milk’s digestion or survival of other bioactive or cryptically bioactive components (e.g., HMOs, lipids, metabolites, extracellular vesicles) remains to be defined.

Given the role of human milk in health (77), defining the impacts of undernutrition and malnutrition on the production, consumption, and digestion of milk is of high importance.

## Development of intestinal immunity

The intestine is considered the largest immune organ in the body (78, 79), containing concentrated regions of lymphoid tissue called gut-associated lymphoid tissue (GALT), as well as myriad innate and adaptive immune cells present throughout the epithelial, submucosal, lamina propria, and muscle layers (79, 80). These cells are tasked with maintaining intestinal homeostasis, preventing inappropriate immune reactions to harmless antigens or microbes (tolerance), while also mounting swift immune reactions to pathogens (activation).

Intestinal macrophages and dendritic cells (DCs) exist in different compartments throughout the small intestine and colon where they perform distinct functions, recently reviewed here (80). Within the lamina propria macrophages participate in barrier maintenance, removal of dying cells, and tolerance of the microbiota (80). Subpopulations of macrophages are also present within Peyers Patches where they present antigen or clear apoptotic immune cells (81–83). Muscularis macrophages are found in the myenteric plexus where they mediate crosstalk with the crosstalk with the enteric nervous system during infection (84). DCs are also present throughout the intestinal lamina propria, including Peyers patches and lymphoid follicles. Here they sample luminal antigens and migrate to mesenteric lymph nodes (85) where they facilitate oral tolerance (86). Notably, draining lymph nodes from each intestinal region harbor DCs with differing levels of tolerance and sensitivity to inflammatory cytokines, for example duodenal DCs are more tolerogenic, while ileal DCs possess more inflammatory cytokine receptors (87). DCs are shaped by their microenvironment, including stromal cell interaction, dietary ligands, and the microbiota. Interestingly, certain subclasses of DCs can affect nutrient uptake, as recently demonstrated for CD11c^+^ cells, which stimulate expression of epithelial lipid transporters (88).

The intestine is also home to a large adaptive immune population. B and T cells are found within small intestinal GALT, which develops alongside the IEC cells *in utero*, with B and T cell clusters visible as early as 14-16 weeks’ gestation (89, 90). These clusters exist as large multi-follicular lymphoid aggregates known as Peyer’s Patches or isolated lymphoid follicles (ILF) of varying sizes throughout the small intestine (SI) (79). ILF are also present within the colon but develop later than small intestinal GALT (90). Both SI and colonic GALT development are influenced by stromal-immune cell crosstalk (90) and house primarily B and T cells (adaptive immunity) as well as dendritic cells to facilitate immune cell priming, mast cells, and granulocytes (91, 92). The intestine is also home to numerous innate immune cells, including the largest population of macrophages in the body, innate lymphoid cells, dendritic cells, and eosinophils. Eosinophils begin to populate the intestine before birth and participate in parasitic and allergic responses (93).

This large and diverse collection of intestinal immune cells, which composes the mucosal immune system, is responsible for surveying the epithelial barrier, protecting against pathogen breach, and maintaining a healthy balance of intestinal microbes. M cells and the follicle-associated epithelium (FAE) (94) which overlay Peyer’s Patches, selectively take up antigens, to facilitate adaptive immunity via DC-mediated antigen presentation (95, 96). For a more detailed discussion of B cells in mucosal immunology, we refer readers to this recent review (97). After antigen exposure, either from luminal contents or deliberately via an oral vaccination, primed B cells clonally expand and migrate between germinal centers. Upon repeat antigen exposure, high-affinity B cell clones are selected. These will be distributed within Peyer’s patches and the lamina propria along the length of the intestine where they produce high affinity secretory immunoglobulin A (sIgA) antibodies against the target antigen as part of the mucosal barrier (92, 98). IgA is one of the primary mediators of the mucosal immune response. sIgA promotes either bacterial clearance through mucus shedding or retention when mucus flow is low (99). sIgA selectivity and specificity shape and regulate the intestinal microbiota (99–101). sIgA also neutralizes pathogen toxins while preventing bacterial proliferation and antigen absorption (102).

## Shaping the intestinal microbiome

Intestinal barrier function, mucosal immunity, and nutrition are intimately connected to the intestinal microbiota. The intestinal tract contains the body’s most abundant and diverse microbial community. The term gut microbiome refers to the community of microbes, their DNA, and byproducts, including the associated metabolome and proteome, which is shaped by host genetics, as well as environmental factors, including mode of birth, diet, antibiotic use, geography, and the host immune system as discussed above (103). Many intestinal microbes coexist within their human host in a mutualistic and beneficial fashion, digesting insoluble fiber (104), modulating host gene expression (105–107), shaping the host immune system (108–111), and providing natural competition for pathogenic organisms within the gut environment (69, 71, 112).

### *In utero* colonization

Whether or not bacteria colonize infants *in utero* remains controversial. A collection of studies detected microbial components in the placenta (113), amniotic fluid (114), umbilical cord blood (115), and meconium (116). However, these studies lack information about the mother’s health, leaving open the argument that the detection of bacteria and bacteria components in the fetal environment could be due to maternal infection or other inflammatory conditions during pregnancy. Although *in utero* microbial colonization is debated, a recent study showed that embryonic IECs can sense short chain fatty acids (SCFAs) produced by the maternal microbiota through G-protein coupled receptors (117), a process that is essential to facilitate the development of fetal EECs. This finding demonstrates that maternal microbial byproducts such as SCFAs can communicate directly with the fetal environment to shape intestinal development, eliminating the need for *in utero* bacteria.

### Postnatal colonization: delivery mode

Bacterial colonization begins at birth and is profoundly influenced by delivery mode (118–120). Vaginally-delivered term infants are introduced to the vaginal microbiota. These first colonizers of the neonatal gut are usually aerobic or facultative bacteria, including *Enterococcus, Streptococcus*, *Prevotella*, *Lactobacillus*, *Bacteroides*, and *Escherichia* (119). Classic culture-based studies between the 1970s and 1980s revealed that the infantile gut microbiota is less complex and has a higher proportion of facultative bacteria than the adult microbiota (121–124). As these bacteria grow, they consume oxygen, making the intestinal environment more hospitable for the proliferation of facultative and anaerobic bacteria, including *Bifidobacterium*, *Clostridium* and *Bacteroides* (122). Once these oxygen-sensitive species establish, the population of aerobic and facultative bacteria decline, and the complexity of the microbiota increases, resulting in a more diverse microbiota (125), closer to that of their adult parents by age five (126, 127).

The microbial communities of infants born by c-section and those born vaginally will eventually converge; however, differences can persist for the first 1-2 years of life (119, 128). In comparison, newborns delivered by cesarean section are deprived of contact with their mother’s gut and vaginal microbiota. They are usually colonized by bacteria associated with the maternal skin and mouth, found on hospital staff, or in the surgical environment (118, 119), delaying the acquisition of *Bacteroides, Bifidobacteria* and *E. coli* (129).

### Feeding mode

Infant nutrition is a significant driver of intestinal microbial colonization and diversity. Human milk contains all the essential nutrients infants need to thrive, including all three primary macronutrients (fats, carbohydrates, proteins), bioactive factors (130) (cytokines, cells, immunoglobulins), immunological factors, prebiotics (e.g., human milk oligosaccharides), and even bacteria, which all contribute to shaping the infant microbiome.

Human milk is a rich source of commensal and mutualistic bacteria (131–136). It is estimated that breastfed infants consume 8 × 10^4^ - 8 × 10^6^ bacteria per 800 mL milk per day, with human milk being the second source of microbes to infants after vaginal birth (131). Human milk contains immunological factors such as maternal IgG and IgA antibodies, which shape the early gut microbiome by dampening mucosal CD4^+^ T helper cell responses and protecting against enteric pathogens (137, 138). Prebiotics in the form of human milk oligosaccharides (HMOs) are another highly abundant and important component of human milk that are indigestible by humans (72, 139) and therefore not present for infant nutrition (140). Rather, HMOs are a rich nutrient source for bacteria, such as *Bifidobacterium longum* ssp *infantis* (141), that shapes the infant microbiome. Early infant gut microbiome is enriched in genes that facilitate lactose utilization found in *Lactobacilli* (142). High levels of *Bifidobacterium* species and bacteria capable of metabolizing HMOs are also found in term infants’ gut (143) and in human milk (119). HMOs are the primary nutrient source in the colon that supports the healthy growth and colonization of these saccharolytic microbiotas, helping to prevent or reduce colonization with specific pathogens (71, 144). In turn, metabolites produced by these bacteria, such as SCFAs, are an important source of energy for enterocytes and key signaling molecules for gut health maintenance (145, 146). There is growing interest in events that shape the milk microbiome, inspired by findings that delivery mode and lactation stage alter the milk microbiota composition (147), suggesting an impact of the physiological labor process, stress, and hormonal signals on the infant microbiota composition (70, 147–151).

### Antibiotic treatment

The use of broad-spectrum antibiotics in infants drastically alters the microbiota. In neonatal intensive care units (NICUs) where antibiotic treatment is common, infants usually acquire a very sparse microbiota almost absent of anaerobes (152, 153). Yeasts, *Enterococcus*, and *Enterobacteriaceae* dominate the microbiota (152, 153). This dysbiosis or imbalance in the gut microbiota places these infants at increased risk of diseases, such as necrotizing enterocolitis (NEC) (154).

## Beneficial roles of the intestinal microbiome on intestinal function

The intestinal microbiome plays an integral role in gut barrier function, including roles in early immune development and immune system priming, as well as direct effects on the IECs, all aimed at restricting bacteria to the luminal compartment of the intestine (155). Commensal microbes compete for space and resources with pathogenic colonizers, which helps reduce disease and can alter the host metabolism (156). The earliest colonizers (*Bifidobacterium*, *Clostridium*, and *Bacteroides* spp.) (125) interact with the mucosa to shape immune system development and intestinal function (157–162). *Lactobacillus* spp. and *Akkermansia muciniphila* proteins stimulate mucus production and strengthen the epithelial barrier (38, 163–167). IEC detect bacterial byproducts or the bacteria themselves through pattern recognition receptors, such as toll-like receptors (TLRs). These signals regulate numerous aspects of the IEC barrier, including mucus and antimicrobial peptide production, tight junctions, and IEC proliferation and differentiation (38, 164, 165, 168, 169).

Bacteria facilitate nutrient digestion by providing the enzymes infants lack for the breakdown of milk glycans (170, 171). The genomes of these bacteria encode a large number of carbohydrate-metabolizing enzymes that are involved in HMO consumption (170, 171), allowing infants to obtain more usable calories from their food. For example, term infants with a diverse community dominant in *Bifidobacterium* and *Bacteroides* have a higher concentration of SCFAs, the end products of fermentation of dietary fibers, than low-birth-weight infants (142). These observations indicate that the microbiota contributes to the digestion of more than 200 different oligosaccharide structures in human milk (172). Moreover, intestinal bacteria possess genes for vitamin synthesis, including vitamin B12 and folate (142).

## Impact of undernutrition on intestinal development and function

The physiology of the intestine in the undernourished state mirrors that of other chronic intestinal diseases, such as Celiac or Crohn’s disease. The villi are sparse and stunted, reducing both the absorptive surface area and the presence of digestive enzymes to mediate nutrient breakdown. This reduced digestive and absorptive surface area likely contributes to failed nutrient absorption and secretory diarrhea following therapeutic feeds. Lactose and glucose malabsorption are highly prevalent among malnourished children (4, 5) and constitute a significant barrier to success with therapeutic feeds. Circumventing these deficiencies through feeding reduced carbohydrate and lactose-free formulations does not improve outcomes (6), indicating that more work is needed to understand the molecular pathophysiology as it impacts nutrient digestion and absorption in malnourished children. There is limited information about the effect of severe undernutrition on the intestinal epithelium.

The effects of undernutrition on the ISC compartment are varied depending on the model. In wasting marmosets, there is no change in ISC or Paneth cell number, but the progenitor pool expanded (173). Several mouse models of undernutrition (1-2% protein) exhibited reduced expression of ISC marker mRNAs (7, 174). These are in sharp contrast to caloric restriction studies in which marked ISC and progenitor expansion occurs (40, 42, 175). In a 28-week, 60% calorie restriction model, Paneth cells also expanded (40); however, this has not been seen in other studies. These expanded progenitor cells are poised for differentiation once nutrient signals direct their specification, but in the case of undernutrition, these signals may be drastically reduced or absent, leading to dysfunction (42, 43, 175, 176). For example, chronic loss of fatty acid oxidation (3 months) in response to undernutrition compromises ISC and progenitor cell function and abolishes the pro-regenerative effects of reduced nutrient consumption (43). How undernutrition alters the ISC niche of human patients remains an open question.

There is also relatively little known about the effects of undernutrition on specific differentiated cells of the intestinal epithelium. Villus atrophy is universally seen across caloric restriction and undernutrition models (7, 40, 42, 173, 175–178), accompanied by a decrease in absorptive enterocytes (40, 42, 173). Goblet cell numbers are reduced in the intestines of mice, rats, and piglet models of undernutrition, as well as mouse models of caloric restriction (40, 42, 173, 179–183). Goblet cell loss leads to reduced mucus (2) leaving the epithelial barrier more susceptible to insults from pathogens or toxins (184). Goblet cell atrophy could result from limited differentiation, dysbiosis (185, 186), or both. Enteroendocrine cells (EEC) produce hormones that regulate growth, hunger, and satiety and are reported as unchanged in wasting marmosets and calorie-restricted mice, although Igarashi et al. found a reduction in EECs in their mouse model of modest (30%) calorie restriction (42). A subset of EECs exhibit reserve stem cell potential (187), which can enhance regeneration after damage (175). The effects of undernutrition on human EECs require further investigation. EECs regulate intestinal growth (17, 18); therefore, dietary modifications influencing EEC form or function could be important for developing targeted dietary therapies for undernutrition. The effect of undernutrition on human tuft cells is unknown, although these cells are drastically expanded in wasting marmosets (173). Given the chemosensory role of tuft cells (20), this expansion may protect the intestinal barrier through immune surveillance and nutrient monitoring.

Undernutrition is characterized by leakiness of the intestinal barrier, which is often measured by urine sugar excretion in human studies (188). Barrier breakdown results from a reduction in tight junction proteins (178) and elevated apoptosis leading to cell loss (177, 178). Reductions in vitamin D signaling may contribute to the disrupted barrier, as seen in inflammatory bowel disease patients (189). Breaches in the barrier allow for luminal bacteria to translocate into the circulation, loss of unabsorbed nutrients through the unrestricted pathway (37, 38), and a loss of absorptive surface area. These effects are exacerbated by decreased bacterial diversity, presumably resulting from the reduction in nutrient intake coupled with changes in the luminal environment (190, 191). The intestinal microbiome is integral to intestinal maturation (190, 192, 193) and its role will be discussed below.

While we do not completely understand the underlying intestinal pathophysiology of undernutrition, several dietary modulations show promise in preclinical models of undernutrition. One study used a mouse model of undernutrition (1% protein diet) to show that bovine milk extracellular vesicles could restore intestinal barrier function after only 4 days of feeding. Although the study did not see differences in growth, the short four-day time point was likely not long enough to appreciate the benefits of a strengthened intestinal barrier (7). Interventions such as bovine milk extracellular vesicles hold promise for reducing the devastating toll of undernutrition on children in the developing world. Another study used germ-free mice colonized with bacteria from a stunted infant to demonstrate that sialylated bovine milk oligosaccharides expand intestinal tuft cells, among other physiological changes. This indicates the importance of understanding the bacteria-substrate interactions and how they may be altered in the presence of dysbiosis to impact growth (192). These studies underscore the importance of the intestine-microbiome relationship on systemic physiology, especially in the context of undernutrition, and how much remains to be discovered in this research realm.

## Adequate nutrition is intimately linked to intestinal immune function

The innate and adaptive immune systems are modulated by nutrient status and key dietary components. Intestinal macrophages and DCs respond to dietary nutrients, indicating the potential for altered functionality in undernutrition. Macrophages are influenced by retinoic acid (194), short chain fatty acids, amino acids (195), and aryl hydrocarbon receptor ligands (196). For example, activation of the aryl hydrocarbon receptor promotes intestinal barrier function and limits inflammation-induced damage (197, 198). DC activity and homing can be shaped by retinoic acid (199), vitamin D (200, 201), and glucose (202) levels.

The presence of enteral nutrients is key to GALT maintenance and function through the maintenance of blood flow through the tissue and continual antigen exposure. Intestinal macrophages are also highly responsive to nutrients and dietary components. In rats, administration of parenteral nutrition, whereby nutrients bypass the digestive system and are administered into the circulation, attenuates immune function in response to a bacterial insult (203, 204) and reduces macrophage regeneration (195). In mice, a 12-hour fast is sufficient to induce GALT atrophy (205) and autopsies of undernourished children reveal a loss of GALT tissue (206). Adequately enterally-nourished hospitalized patients with preserved mucosal immunity exhibit reduced infections and faster discharge times compared to patients experiencing undernutrition or fed parenterally (207–209), emphasizing the importance of enteral nutrition in critically ill patients.

In addition to GALT tissue loss, undernourished children secrete less sIgA (206, 210, 211) or secrete IgA that binds and retains pathogenic bacteria capable of disrupting the intestinal barrier and causing weight loss when transplanted into mice (210). sIgA function is intimately linked to nutrition (101, 210). sIgA selectivity and specificity are shaped by interactions between the IgA molecule and bacterial surface glycans. These bacterial glycans are influenced by the host’s diet and mediate differential bacterial retention or shedding based on binding specificity (101). Retention of beneficial bacteria has health benefits, such as enhanced barrier function (212) and shaping the overall microbiome ecosystem (71), while retaining pathogenic bacteria can lead to disease (210). Imbalances in the microbial community increase the risk of bacterial infections, which are exceedingly common in malnourished children (213). sIgA is therefore a critical mediator of intestinal microbial balance and, when disrupted, magnifies the adverse effects of under or malnutrition by promoting pathogenic colonization, reducing commensal colonization, and exacerbating weight loss and barrier defects concomitant with low nutrient states (101, 210).

Collectively, this suggests that nutrient status is linked to immune tolerance and the ability to mount appropriate immune responses indicating that undernutrition likely hampers immune function.

## Impact of undernutrition on the intestinal microbiome and host

The symbiotic relationship between IEC, barrier function, and the associated microbiome is intimately impacted by nutrition (214–218). Numerous studies over the last decade illustrate that undernutrition is linked to reduced microbial diversity and microbiome immaturity (111, 190, 210, 219–221) and that this “malnourished microbiome” is a major contributor to undernutrition phenotypes as these can be transferred to animal models with resulting impaired growth despite the provision of adequate calories (111, 210, 221). Undernutrition is also associated with alterations in bacterial retention due to changes in bacteria glycans and IgA affinity, which can affect the intestinal barrier (101). In a 2014 study, microbiota maturity indices were used to measure postnatal microbiota development in humans from birth up to 24 months, giving rise to a means to classify malnutritional states (190). They used machine-learning approaches to identify a set of “age-discriminatory” taxa that defines “microbiota age.” The model was then verified in a second cohort of Bangladeshi children and a cohort of healthy children in Malawi, suggesting that it may be used universally as a reference for normal intestinal microbial ecology, and for comparison with a malnourished microbiome.

The microbiota of undernourished children is less efficient at energy extraction from dietary nutrients, resulting in changes in microbial metabolite production (222). Microbial metabolites are critical regulators of intestinal barrier function (223), therefore dysbiosis can exacerbate barrier leakiness and inflammation. Additionally, microbial metabolites alter the host epigenome, which can have long-term impacts on host health and physiology, suggesting a potential mechanism for the sustained adverse effects of undernutrition even after proper nutrients are provided (111, 222).

Loss of various dietary components, including protein and antioxidants, can severely impact microbiota composition and in turn barrier function. A metanalysis across malnourished children at five geographic sites suggests that loss of dietary antioxidants, such as vitamins C and E and carotenoids, can alter the redox potential of the gut, causing dysbiosis (224). They observed a loss of specific anaerobic bacterial species, such as those from the Bacteroidetes or Eubacteriaceae families, and increases in aerotolerant bacteria like *Escherichia coli, Enterococcus faecalis*, and *Staphylococcus aureus*, which are considered common pathogens (224), indicating the importance of dietary antioxidants in maintaining intestinal bacterial diversity and reducing risk of enteric infection.

The ability to induce a sustained shift from an immature, malnourished microbiome to a healthier, more diverse set of bacteria is a major focus of current research (193, 225, 226). Simply increasing nutrient intake is insufficient to permanently shift the microbiome away from a malnourished state to a more healthy and diverse population that maximizes energy extraction and beneficial metabolite production. Recent work indicates that microbiota-directed food interventions for undernourished children may improve long-term health outcomes in undernutrition (225, 226). These interventions provide foods that not only offer adequate energy but also support a shift in bacterial taxa away from the malnourished microbiome. In an interventional study, Chen et al. (193) administered microbiota-directed complementary food prototype (MDCF-2) or the standard ready-to-use supplementary food (RUSF) to Bangladeshi children with moderate acute undernutrition for three months and monitored growth, plasma protein biomarkers, and fecal bacteria one month following the intervention. The results indicated that the MDCF-2 diet promoted growth and was linked to circulating proteins associated with bone growth and neurodevelopment, as well as a more substantial restoration of the intestinal microbiota, indicating the importance of nutrients that mediate healthy shifts in the microbiome and not simply those that add calories (193). Although these findings need to be validated in more sites and at a later time post-treatment, they show promise for improved therapeutic diets for undernutrition. Maintaining this sustained shift to a mature and diverse gut microbiome is critical to preventing long-term negative consequences of undernutrition, such as neurodevelopmental delays and the development of obesity and metabolic disease later in life (218, 227).

Modulating the intestinal microbiome in undernutrition has the potential to improve intestinal barrier and immune function, as well as host energy extraction to improve long-term outcomes.

## Limitations of current models and future directions

To combat malnutrition successfully, particularly undernutrition, we need a more detailed understanding of the associated underlying intestinal pathophysiology and intestinal microbiome derangements. Presently, most non-interventional undernutrition studies are conducted in mice or rats using reduced or very low protein diets in isolation (7, 174, 176, 177, 228) or in combination with a germ-free background (229). While these models exhibit some similarities to human undernutrition, especially reduced growth, intestinal epithelial villus stunting, and reduced IEC proliferation, they do not always mimic the physiologic or morphologic changes (27). Additionally, the intestines of mice and rats are immature and permeable at birth (27, 230), whereas the human neonatal intestine is relatively mature and only selectively permeable, more similar to the intestines of young pigs (183, 231) or guinea pigs (232–235). Notably, most studies of GALT took place in murine models where development and composition are distinct from humans in several important ways. Murine ILF develop early in prenatal development (236) and consist almost entirely of B cells, whereas human ILF develop shortly before and after birth and contain proportionally more T cells (237–239). Therefore, modeling undernutrition in animal models should account for these intestinal differences, recognizing the caveats of using less mainstream species. Non-human primate models of undernutrition could address the shortcomings of more distantly-related models, with a potential for more relevant and detailed understanding of the impacts of the effects on the intestinal epithelium and microbiome, but cost is a factor in such research.

Human enteroid culture could circumvent many of these challenges and allow for testing therapies and delineating pathophysiological mechanisms within malnourished human tissue. Enteroids are a rapidly emerging model system for studying complex intestinal diseases and interactions (240), including necrotizing enterocolitis (241, 242), bacterial or viral infection (243–249) and fundamental IEC dynamics (250, 251). The ability to model undernutrition *ex vivo* using human tissue could accelerate progress in reducing and eliminating the devastating consequences of undernutrition.

Important unanswered questions include (1) how specific nutrients or dietary components such as vitamin D, substrates for the aryl hydrocarbon receptor, protein, carbohydrates, or even food-derived extracellular vesicles impact intestinal cell populations in the malnourished intestine; (2) how nutrients alter barrier function in the malnourished intestine; (3) the timing of physiological changes to the intestine in response to undernutrition; (4) what constitutes the composition of a healthy gut microbiota in well-fed individuals; and (5) which core gut microbes will lead to the most favorable health outcomes. To tackle these questions and more, we need improved models to study undernutrition, including those that incorporate the microbiome (252). Furthermore, we need to understand how maternal health and the microbiome prior to and during pregnancy shape the foundations of infant gut development and health, and how the dynamic interactions of the mother-child dyad influence the same through long-term longitudinal studies.

## Author contributions

The corresponding author had the responsibility for the decision to submit the manuscript. All authors assisted in manuscript preparation and all authors read and approved the final version of the manuscript.

## References

[1] WHO. Malnutrition (2021). WHO. Available at: https://www.who.int/news-room/fact-sheets/detail/malnutrition (Accessed Feb 1 2022).

[2] Attia S Feenstra M Swain N Cuesta M Bandsma RHJ. Starved guts: morphologic and functional intestinal changes in malnutrition. J Pediatr Gastroenterol Nutr (2017) 65:491–5. doi: 10.1097/MPG.0000000000001629 28489672

[3] Bhutta ZA Das JK Rizvi A Gaffey MF Walker N Horton S. Evidence-based interventions for improvement of maternal and child nutrition: what can be done and at what cost? Lancet (2013) 382:452–77. doi: 10.1016/S0140-6736(13)60996-4 23746776

[4] Bandsma RH Spoelstra MN Mari A Mendel M Van Rheenen PF Senga E. Impaired glucose absorption in children with severe malnutrition. J Pediatr (2011) 158:282–287 e281. doi: 10.1016/j.jpeds.2010.07.048 20843523

[5] Kvissberg MA Dalvi PS Kerac M Voskuijl W Berkley JA Priebe MG. Carbohydrate malabsorption in acutely malnourished children and infants: a systematic review. Nutr Rev (2016) 74:48–58. doi: 10.1093/nutrit/nuv058 26578625 PMC4684688

[6] Bandsma RHJ Voskuijl W Chimwezi E Fegan G Briend A Thitiri J. A reduced-carbohydrate and lactose-free formulation for stabilization among hospitalized children with severe acute malnutrition: a double-blind, randomized controlled trial. PloS Med (2019) 16:e1002747. doi: 10.1371/journal.pmed.1002747 30807589 PMC6390989

[7] Maghraby MK Li B Chi L Ling C Benmoussa A Provost P. Extracellular vesicles isolated from milk can improve gut barrier dysfunction induced by malnutrition. Sci Rep (2021) 11:7635. doi: 10.1038/s41598-021-86920-w 33828139 PMC8026962

[8] Soderholm AT Pedicord VA. Intestinal epithelial cells: at the interface of the microbiota and mucosal immunity. Immunology (2019) 158:267–80. doi: 10.1111/imm.13117 PMC6856932 31509239

[9] Helander HF Fandriks L. Surface area of the digestive tract - revisited. Scand J Gastroenterol (2014) 49:681–9. doi: 10.3109/00365521.2014.898326 24694282

[10] Chin AM Hill DR Aurora M Spence JR. Morphogenesis and maturation of the embryonic and postnatal intestine. Semin Cell Dev Biol (2017) 66:81–93. doi: 10.1016/j.semcdb.2017.01.011 28161556 PMC5487846

[11] Beumer J Clevers H. Cell fate specification and differentiation in the adult mammalian intestine. Nat Rev Mol Cell Biol (2021) 22:39–53. doi: 10.1038/s41580-020-0278-0 32958874

[12] Van Der Flier LG Clevers H. Stem cells, self-renewal, and differentiation in the intestinal epithelium. Annu Rev Physiol (2009) 71:241–60. doi: 10.1146/annurev.physiol.010908.163145 18808327

[13] Cheng H Leblond CP. Origin, differentiation and renewal of the four main epithelial cell types in the mouse small intestine. i. columnar cell. Am J Anat (1974) 141:461–79. doi: 10.1002/aja.1001410403 4440632

[14] Barker N Van Es JH Kuipers J Kujala P Van Den Born M Cozijnsen M. Identification of stem cells in small intestine and colon by marker gene Lgr5. Nature (2007) 449:1003–7. doi: 10.1038/nature06196 17934449

[15] Basak O Van De Born M Korving J Beumer J van der Elst S Van Es JH. Mapping early fate determination in Lgr5+ crypt stem cells using a novel Ki67-RFP allele. EMBO J (2014) 33:2057–68. doi: 10.15252/embj.201488017 PMC4195772 25092767

[16] Antoni L Nuding S Weller D Gersemann M Ott G Wehkamp J. Human colonic mucus is a reservoir for antimicrobial peptides. J Crohns Colitis (2013) 7:e652–664. doi: 10.1016/j.crohns.2013.05.006 23787054

[17] Dahly EM Gillingham MB Guo Z Murali SG Nelson DW Holst JJ. Role of luminal nutrients and endogenous GLP-2 in intestinal adaptation to mid-small bowel resection. Am J Physiol Gastrointest Liver Physiol (2003) 284:G670–682. doi: 10.1152/ajpgi.00293.2002 12505881

[18] Koopmann MC Chen X Holst JJ Ney DM. Sustained glucagon-like peptide-2 infusion is required for intestinal adaptation, and cessation reverses increased cellularity in rats with intestinal failure. Am J Physiol Gastrointest Liver Physiol (2010) 299:G1222–1230. doi: 10.1152/ajpgi.00367.2010 PMC3006245 20864657

[19] Koehler JA Baggio LL Yusta B Longuet C Rowland KJ Cao X. GLP-1R agonists promote normal and neoplastic intestinal growth through mechanisms requiring Fgf7. Cell Metab (2015) 21:379–91. doi: 10.1016/j.cmet.2015.02.005 25738454

[20] Von Moltke J. Physiology of the gastrointestinal tract (Sixth edition). Academic Press (2018) p. 721–33. doi: 10.1016/B978-0-12-809954-4.00031-1

[21] Bjerknes M Cheng H. The stem-cell zone of the small intestinal epithelium. I. Evidence from Paneth cells in the adult mouse. Am J Anat (1981) 160:51–63. doi: 10.1002/aja.1001600105 7211716

[22] Bevins CL Salzman NH. Paneth cells, antimicrobial peptides and maintenance of intestinal homeostasis. Nat Rev Microbiol (2011) 9:356–68. doi: 10.1038/nrmicro2546 21423246

[23] Axelsson I Jakobsson I Lindberg T Polberger S Benediktsson B Raiha N. Macromolecular absorption in preterm and term infants. Acta Paediatr Scand (1989) 78:532–7. doi: 10.1111/j.1651-2227.1989.tb17932.x 2782068

[24] Catassi C Bonucci A Coppa GV Carlucci A Giorgi PL. Intestinal permeability changes during the first month: effect of natural versus artificial feeding. J Pediatr Gastroenterol Nutr (1995) 21:383–6. doi: 10.1097/00005176-199511000-00003 8583288

[25] Taylor SN Basile LA Ebeling M Wagner CL. Intestinal permeability in preterm infants by feeding type: mother's milk versus formula. Breastfeed Med (2009) 4:11–5. doi: 10.1089/bfm.2008.0114 PMC2932544 19196035

[26] Humphrey E Claud E. Impact of microbes on the intestinal development of the preterm infant. In: Sun J Dudeja PK, editors. Mechanisms underlying host-microbiome interactions in pathophysiology of human diseases. Boston, MA: Springer US (2018). p. 1–33.

[27] Westrom B Arevalo Sureda E Pierzynowska K Pierzynowski SG Perez-Cano FJ. The immature gut barrier and its importance in establishing immunity in newborn mammals. Front Immunol (2020) 11:1153. doi: 10.3389/fimmu.2020.01153 32582216 PMC7296122

[28] France MM Turner JR. The mucosal barrier at a glance. J Cell Sci (2017) 130:307–14. doi: 10.1242/jcs.193482 PMC5278669 28062847

[29] Francis SA Kelly JM Mccormack J Rogers RA Lai J Schneeberger EE. Rapid reduction of MDCK cell cholesterol by methyl-beta-cyclodextrin alters steady state transepithelial electrical resistance. Eur J Cell Biol (1999) 78:473–84. doi: 10.1016/S0171-9335(99)80074-0 10472800

[30] Green KJ Simpson CL. Desmosomes: new perspectives on a classic. J Invest Dermatol (2007) 127:2499–515. doi: 10.1038/sj.jid.5701015 17934502

[31] Baum B Georgiou M. Dynamics of adherens junctions in epithelial establishment, maintenance, and remodeling. J Cell Biol (2011) 192:907–17. doi: 10.1083/jcb.201009141 PMC3063136 21422226

[32] Simon DB Lu Y Choate KA Velazquez H Al-Sabban E Praga M. Paracellin-1, a renal tight junction protein required for paracellular Mg2+ resorption. Science (1999) 285:103–6. doi: 10.1126/science.285.5424.103 10390358

[33] Amasheh S Meiri N Gitter AH Schoneberg T Mankertz J Schulzke JD. Claudin-2 expression induces cation-selective channels in tight junctions of epithelial cells. J Cell Sci (2002) 115:4969–76. doi: 10.1242/jcs.00165 12432083

[34] Colegio OR Van Itallie C Rahner C Anderson JM. Claudin extracellular domains determine paracellular charge selectivity and resistance but not tight junction fibril architecture. Am J Physiol Cell Physiol (2003) 284:C1346–1354. doi: 10.1152/ajpcell.00547.2002 12700140

[35] Watson CJ Hoare CJ Garrod DR Carlson GL Warhurst G. Interferon-gamma selectively increases epithelial permeability to large molecules by activating different populations of paracellular pores. J Cell Sci (2005) 118:5221–30. doi: 10.1242/jcs.02630 16249235

[36] Van Itallie CM Holmes J Bridges A Gookin JL Coccaro MR Proctor W. The density of small tight junction pores varies among cell types and is increased by expression of claudin-2. J Cell Sci (2008) 121:298–305. doi: 10.1242/jcs.021485 18198187

[37] Aihara E Matthis AL Karns RA Engevik KA Jiang P Wang J. Epithelial regeneration after gastric ulceration causes prolonged cell-type alterations. Cell Mol Gastroenterol Hepatol (2016) 2:625–47. doi: 10.1016/j.jcmgh.2016.05.005 PMC5042868 27766298

[38] Yu J Ordiz MI Stauber J Shaikh N Trehan I Barnell E. Environmental enteric dysfunction includes a broad spectrum of inflammatory responses and epithelial repair processes. Cell Mol Gastroenterol Hepatol (2016) 2:158–174 e151. doi: 10.1016/j.jcmgh.2015.12.002 26973864 PMC4769221

[39] Secor SM Stein ED Diamond J. Rapid upregulation of snake intestine in response to feeding: a new model of intestinal adaptation. Am J Physiol (1994) 266:G695–705. doi: 10.1152/ajpgi.1994.266.4.G695 8179004

[40] Yilmaz OH Katajisto P Lamming DW Gultekin Y Bauer-Rowe KE Sengupta S. mTORC1 in the Paneth cell niche couples intestinal stem-cell function to calorie intake. Nature (2012) 486:490–5. doi: 10.1038/nature11163 PMC3387287 22722868

[41] Zhou Y Rychahou P Wang Q Weiss HL Evers BM. TSC2/mTORC1 signaling controls Paneth and goblet cell differentiation in the intestinal epithelium. Cell Death Dis (2015) 6:e1631. doi: 10.1038/cddis.2014.588 25654764 PMC4669793

[42] Igarashi M Guarente L. mTORC1 and SIRT1 cooperate to foster expansion of gut adult stem cells during calorie restriction. Cell (2016) 166:436–50. doi: 10.1016/j.cell.2016.05.044 27345368

[43] Mihaylova MM Cheng CW Cao AQ Tripathi S Mana MD Bauer-Rowe KE. Fasting activates fatty acid oxidation to enhance intestinal stem cell function during homeostasis and aging. Cell Stem Cell (2018) 22:769–778 e764. doi: 10.1016/j.stem.2018.04.001 29727683 PMC5940005

[44] Beyaz S Mana MD Yilmaz OH. High-fat diet activates a PPAR-delta program to enhance intestinal stem cell function. Cell Stem Cell (2021) 28:598–9. doi: 10.1016/j.stem.2021.03.001 PMC8875728 33798420

[45] Rodriguez-Colman MJ Schewe M Meerlo M Stigter E Gerrits J Pras-Raves M. Interplay between metabolic identities in the intestinal crypt supports stem cell function. Nature (2017) 543:424–7. doi: 10.1038/nature21673 28273069

[46] Cheng CW Biton M Haber AL Gunduz N Eng G Gaynor LT. Ketone body signaling mediates intestinal stem cell homeostasis and adaptation to diet. Cell (2019) 178:1115–1131 e1115. doi: 10.1016/j.cell.2019.07.048 31442404 PMC6732196

[47] Mah AT Van Landeghem L Gavin HE Magness ST Lund PK. Impact of diet-induced obesity on intestinal stem cells: hyperproliferation but impaired intrinsic function that requires insulin/IGF1. Endocrinology (2014) 155:3302–14. doi: 10.1210/en.2014-1112 PMC4138564 24914941

[48] Mana MD Hussey AM Tzouanas CN Imada S Barrera Millan Y Bahceci D. High-fat diet-activated fatty acid oxidation mediates intestinal stemness and tumorigenicity. Cell Rep (2021) 35:109212. doi: 10.1016/j.celrep.2021.109212 34107251 PMC8258630

[49] Wang D Fu L Wei J Xiong Y Dubois RN. PPARdelta mediates the effect of dietary fat in promoting colorectal cancer metastasis. Cancer Res (2019) 79(17):4480–90. doi: 10.1158/0008-5472.CAN-19-0384 PMC6726501 31239272

[50] Chen SW Wang PY Zhu J Chen GW Zhang JL Chen ZY. Protective effect of 1,25-dihydroxyvitamin d3 on lipopolysaccharide-induced intestinal epithelial tight junction injury in caco-2 cell monolayers. Inflammation (2015) 38:375–83. doi: 10.1007/s10753-014-0041-9 25344656

[51] Peregrina K Houston M Daroqui C Dhima E Sellers RS Augenlicht LH. Vitamin D is a determinant of mouse intestinal Lgr5 stem cell functions. Carcinogenesis (2015) 36:25–31. doi: 10.1093/carcin/bgu221 25344836 PMC4303796

[52] Metidji A Omenetti S Crotta S Li Y Nye E Ross E. The environmental sensor AHR protects from inflammatory damage by maintaining intestinal stem cell homeostasis and barrier integrity. Immunity (2018) 49:353–362 e355. doi: 10.1016/j.immuni.2018.07.010 30119997 PMC6104739

[53] Qiu J Heller JJ Guo X Chen ZM Fish K Fu YX. The aryl hydrocarbon receptor regulates gut immunity through modulation of innate lymphoid cells. Immunity (2012) 36:92–104. doi: 10.1016/j.immuni.2011.11.011 22177117 PMC3268875

[54] Zelante T Iannitti RG Cunha C De Luca A Giovannini G Pieraccini G. Tryptophan catabolites from microbiota engage aryl hydrocarbon receptor and balance mucosal reactivity via interleukin-22. Immunity (2013) 39:372–85. doi: 10.1016/j.immuni.2013.08.003 23973224

[55] Meng D Sommella E Salviati E Campiglia P Ganguli K Djebali K. Indole-3-lactic acid, a metabolite of tryptophan, secreted by bifidobacterium longum subspecies infantis is anti-inflammatory in the immature intestine. Pediatr Res (2020) 88:209–17. doi: 10.1038/s41390-019-0740-x PMC7363505 31945773

[56] Lu P Yamaguchi Y Fulton WB Wang S Zhou Q Jia H. Maternal aryl hydrocarbon receptor activation protects newborns against necrotizing enterocolitis. Nat Commun (2021) 12:1042. doi: 10.1038/s41467-021-21356-4 33589625 PMC7884836

[57] Nolan LS Mihi B Agrawal P Gong Q Rimer JM Bidani SS. Indole-3-Carbinol-Dependent aryl hydrocarbon receptor signaling attenuates the inflammatory response in experimental necrotizing enterocolitis. Immunohorizons (2021) 5:193–209. doi: 10.4049/immunohorizons.2100018 33906960 PMC8173979

[58] Sankar MJ Sinha B Chowdhury R Bhandari N Taneja S Martines J. Optimal breastfeeding practices and infant and child mortality: a systematic review and meta-analysis. Acta Paediatr (2015) 104:3–13. doi: 10.1111/apa.13147 26249674

[59] Belfort MB Ramel SE. NICU diet, physical growth and nutrient accretion, and preterm infant brain development. Neoreviews (2019) 20:e385–96. doi: 10.1542/neo.20-7-e385 31261105

[60] Belfort MB Inder TE. Human milk and preterm infant brain development: a narrative review. Clin Ther (2022) 44:612–21. doi: 10.1016/j.clinthera.2022.02.011 PMC9133155 35307209

[61] Meek JY Noble L Section On B. Policy statement: breastfeeding and the use of human milk. Pediatrics (2022) 150(1). doi: 10.1542/peds.2022-057988 35921640

[62] Forsum E Lonnerdal B. Effect of protein intake on protein and nitrogen composition of breast milk. Am J Clin Nutr (1980) 33:1809–13. doi: 10.1093/ajcn/33.8.1809 7405883

[63] Minato T Nomura K Asakura H Aihara A Hiraike H Hino Y. Maternal undernutrition and breast milk macronutrient content are not associated with weight in breastfed infants at 1 and 3 months after delivery. Int J Environ Res Public Health (2019) 16(18). doi: 10.3390/ijerph16183315 PMC6765925 31505822

[64] Rockers P Sharda A Shet A. Maternal malnutrition, breastfeeding, and child inflammation in India (P11-025-19). Curr Dev Nutr (2019) 3(Supplement_1):nzz048.P11-25-19. doi: 10.1093/cdn/nzz048.P11-025-19

[65] Ahmed T Hossain M Sanin KI. Global burden of maternal and child undernutrition and micronutrient deficiencies. Ann Nutr Metab (2012) 61 Suppl 1:8–17. doi: 10.1159/000345165 23343943

[66] Christian P Mullany LC Hurley KM Katz J Black RE. Nutrition and maternal, neonatal, and child health. Semin Perinatol (2015) 39:361–72. doi: 10.1053/j.semperi.2015.06.009 26166560

[67] Andres S Scottoline B Good M. Shaping infant development from the inside out: bioactive factors in human milk. Semin Perinatol (2022) 47(1):151690. doi: 10.1016/j.semperi.2022.151690 36581527 PMC9974576

[68] Patra K Hamilton M Johnson TJ Greene M Dabrowski E Meier PP. NICU human milk dose and 20-month neurodevelopmental outcome in very low birth weight infants. Neonatology (2017) 112:330–6. doi: 10.1159/000475834 PMC5683911 28768286

[69] Liu Z Roy NC Guo Y Jia H Ryan L Samuelsson L. Human breast milk and infant formulas differentially modify the intestinal microbiota in human infants and host physiology in rats. J Nutr (2016) 146:191–9. doi: 10.3945/jn.115.223552 26674765

[70] Murphy K Curley D O'callaghan TF O'shea C-A Dempsey EM O'toole PW. The composition of human milk and infant faecal microbiota over the first three months of life: a pilot study. Sci Rep (2017) 7:40597. doi: 10.1038/srep40597 28094284 PMC5240090

[71] Duar RM Henrick BM Casaburi G Frese SA. Integrating the ecosystem services framework to define dysbiosis of the breastfed infant gut: the role of b. infantis and human milk oligosaccharides. Front Nutr (2020) 7:33. doi: 10.3389/fnut.2020.00033 32346537 PMC7171047

[72] Walsh C Lane JA Van Sinderen D Hickey RM. Human milk oligosaccharides: shaping the infant gut microbiota and supporting health. J Funct Foods (2020) 72:104074. doi: 10.1016/j.jff.2020.104074 32834834 PMC7332462

[73] Lonnerdal B. Nutritional and physiologic significance of human milk proteins. Am J Clin Nutr (2003) 77:1537S–43S. doi: 10.1093/ajcn/77.6.1537S 12812151

[74] Chen Y Patel A Meier PP Fantuzzi G. Digested early preterm human milk suppresses tumor necrosis factor-induced inflammation and cytotoxicity in intestinal epithelial cells. J Pediatr Gastroenterol Nutr (2018) 66:e153–7. doi: 10.1097/MPG.0000000000001932 29470288

[75] Liang N Beverly RL Scottoline BP Dallas DC. Peptides derived from *In vitro* and *In vivo* digestion of human milk are immunomodulatory in THP-1 human macrophages. J Nutr (2022) 152:331–42. doi: 10.1093/jn/nxab350 PMC8754566 34601601

[76] Beverly RL Woonnimani P Scottoline BP Lueangsakulthai J Dallas DC. Peptides from the intestinal tract of breast milk-fed infants have antimicrobial and bifidogenic activity. Int J Mol Sci (2021) 22(5). doi: 10.3390/ijms22052377 PMC7956819 33673498

[77] Victora CG Bahl R Barros AJ Franca GV Horton S Krasevec J. Breastfeeding in the 21st century: epidemiology, mechanisms, and lifelong effect. Lancet (2016) 387:475–90. doi: 10.1016/S0140-6736(15)01024-7 26869575

[78] Fukatsu K Kudsk KA. Nutrition and gut immunity. Surg Clin North Am (2011) 91:755–770, vii. doi: 10.1016/j.suc.2011.04.007 21787966 PMC3144400

[79] Mowat AM Agace WW. Regional specialization within the intestinal immune system. Nat Rev Immunol (2014) 14(5):667–85. doi: 10.1038/nri3738 25234148

[80] Filardy AA Ferreira JRM Rezende RM Kelsall BL Oliveira RP. The intestinal microenvironment shapes macrophage and dendritic cell identity and function. Immunol Lett (2023) 253:41–53. doi: 10.1016/j.imlet.2023.01.003 36623708 PMC9907447

[81] Lelouard H Henri S De Bovis B Mugnier B Chollat-Namy A Malissen B. Pathogenic bacteria and dead cells are internalized by a unique subset of peyer's patch dendritic cells that express lysozyme. Gastroenterology (2010) 138:173–184 e171-173. doi: 10.1053/j.gastro.2009.09.051 19800337

[82] Rahman ZS Shao WH Khan TN Zhen Y Cohen PL. Impaired apoptotic cell clearance in the germinal center by mer-deficient tingible body macrophages leads to enhanced antibody-forming cell and germinal center responses. J Immunol (2010) 185:5859–68. doi: 10.4049/jimmunol.1001187 PMC3563244 20952679

[83] Bonnardel J Da Silva C Henri S Tamoutounour S Chasson L Montanana-Sanchis F. Innate and adaptive immune functions of peyer's patch monocyte-derived cells. Cell Rep (2015) 11:770–84. doi: 10.1016/j.celrep.2015.03.067 25921539

[84] Muller PA Koscso B Rajani GM Stevanovic K Berres ML Hashimoto D. Crosstalk between muscularis macrophages and enteric neurons regulates gastrointestinal motility. Cell (2014) 158:300–13. doi: 10.1016/j.cell.2014.04.050 PMC4149228 25036630

[85] Luciani C Hager FT Cerovic V Lelouard H. Dendritic cell functions in the inductive and effector sites of intestinal immunity. Mucosal Immunol (2022) 15:40–50. doi: 10.1038/s41385-021-00448-w 34465895

[86] Worbs T Bode U Yan S Hoffmann MW Hintzen G Bernhardt G. Oral tolerance originates in the intestinal immune system and relies on antigen carriage by dendritic cells. J Exp Med (2006) 203:519–27. doi: 10.1084/jem.20052016 PMC2118247 16533884

[87] Esterhazy D Loschko J London M Jove V Oliveira TY Mucida D. Classical dendritic cells are required for dietary antigen-mediated induction of peripheral t(reg) cells and tolerance. Nat Immunol (2016) 17:545–55. doi: 10.1038/ni.3408 PMC4837106 27019226

[88] Guendel F Kofoed-Branzk M Gronke K Tizian C Witkowski M Cheng HW. Group 3 innate lymphoid cells program a distinct subset of IL-22BP-Producing dendritic cells demarcating solitary intestinal lymphoid tissues. Immunity (2020) 53:1015–1032 e1018. doi: 10.1016/j.immuni.2020.10.012 33207209

[89] Braegger CP Spencer J Macdonald TT. Ontogenetic aspects of the intestinal immune system in man. Int J Clin Lab Res (1992) 22:1–4. doi: 10.1007/BF02591385 1633313

[90] Fawkner-Corbett D Antanaviciute A Parikh K Jagielowicz M Geros AS Gupta T. Spatiotemporal analysis of human intestinal development at single-cell resolution. Cell (2021) 184:810–826 e823. doi: 10.1016/j.cell.2020.12.016 33406409 PMC7864098

[91] Morbe UM Jorgensen PB Fenton TM Von Burg N Riis LB Spencer J. Human gut-associated lymphoid tissues (GALT); diversity, structure, and function. Mucosal Immunol (2021) 14:793–802. doi: 10.1038/s41385-021-00389-4 33753873

[92] Quiroz-Olguin G Gutierrez-Salmean G Posadas-Calleja JG Padilla-Rubio MF Serralde-Zuniga AE. The effect of enteral stimulation on the immune response of the intestinal mucosa and its application in nutritional support. Eur J Clin Nutr (2021) 75:1533–9. doi: 10.1038/s41430-021-00877-7 33608653

[93] Carter PB Collins FM. The route of enteric infection in normal mice. J Exp Med (1974) 139:1189–203. doi: 10.1084/jem.139.5.1189 PMC2139651 4596512

[94] Jorgensen PB Fenton TM Morbe UM Riis LB Jakobsen HL Nielsen OH. Identification, isolation and analysis of human gut-associated lymphoid tissues. Nat Protoc (2021) 16:2051–67. doi: 10.1038/s41596-020-00482-1 33619391

[95] Hase K Kawano K Nochi T Pontes GS Fukuda S Ebisawa M. Uptake through glycoprotein 2 of FimH(+) bacteria by M cells initiates mucosal immune response. Nature (2009) 462:226–30. doi: 10.1038/nature08529 19907495

[96] Pabst O. New concepts in the generation and functions of IgA. Nat Rev Immunol (2012) 12:821–32. doi: 10.1038/nri3322 23103985

[97] Spencer J Bemark M. Human intestinal b cells in inflammatory diseases. Nat Rev Gastroenterol Hepatol (2023) 20:254–65. doi: 10.1038/s41575-023-00755-6 36849542

[98] Bergqvist P Stensson A Hazanov L Holmberg A Mattsson J Mehr R. Re-utilization of germinal centers in multiple Peyer's patches results in highly synchronized, oligoclonal, and affinity-matured gut IgA responses. Mucosal Immunol (2013) 6:122–35. doi: 10.1038/mi.2012.56 22785230

[99] Mcloughlin K Schluter J Rakoff-Nahoum S Smith AL Foster KR. Host selection of microbiota via differential adhesion. Cell Host Microbe (2016) 19:550–9. doi: 10.1016/j.chom.2016.02.021 27053168

[100] Moor K Diard M Sellin ME Felmy B Wotzka SY Toska A. High-avidity IgA protects the intestine by enchaining growing bacteria. Nature (2017) 544:498–502. doi: 10.1038/nature22058 28405025

[101] Donaldson GP Ladinsky MS Yu KB Sanders JG Yoo BB Chou WC. Gut microbiota utilize immunoglobulin a for mucosal colonization. Science (2018) 360:795–800. doi: 10.1126/science.aaq0926 29724905 PMC5973787

[102] Woof JM Kerr MA. The function of immunoglobulin a in immunity. J Pathol (2006) 208:270–82. doi: 10.1002/path.1877 16362985

[103] Gill SR Pop M Deboy RT Eckburg PB Turnbaugh PJ Samuel BS. Metagenomic analysis of the human distal gut microbiome. Science (2006) 312:1355–9. doi: 10.1126/science.1124234 PMC3027896 16741115

[104] Kovatcheva-Datchary P Nilsson A Akrami R Lee YS De Vadder F Arora T. Dietary fiber-induced improvement in glucose metabolism is associated with increased abundance of prevotella. Cell Metab (2015) 22:971–82. doi: 10.1016/j.cmet.2015.10.001 26552345

[105] Ley RE Hamady M Lozupone C Turnbaugh PJ Ramey RR Bircher JS. Evolution of mammals and their gut microbes. Science (2008) 320:1647–51. doi: 10.1126/science.1155725 PMC2649005 18497261

[106] Muegge BD Kuczynski J Knights D Clemente JC Gonzalez A Fontana L. Diet drives convergence in gut microbiome functions across mammalian phylogeny and within humans. Science (2011) 332:970–4. doi: 10.1126/science.1198719 PMC3303602 21596990

[107] Walker AW Ince J Duncan SH Webster LM Holtrop G Ze X. Dominant and diet-responsive groups of bacteria within the human colonic microbiota. ISME J (2011) 5:220–30. doi: 10.1038/ismej.2010.118 PMC3105703 20686513

[108] Wu GD Chen J Hoffmann C Bittinger K Chen YY Keilbaugh SA. Linking long-term dietary patterns with gut microbial enterotypes. Science (2011) 334:105–8. doi: 10.1126/science.1208344 PMC3368382 21885731

[109] Cotillard A Kennedy SP Kong LC Prifti E Pons N Le Chatelier E. Dietary intervention impact on gut microbial gene richness. Nature (2013) 500:585–8. doi: 10.1038/nature12480 23985875

[110] David LA Maurice CF Carmody RN Gootenberg DB Button JE Wolfe BE. Diet rapidly and reproducibly alters the human gut microbiome. Nature (2014) 505:559–63. doi: 10.1038/nature12820 PMC3957428 24336217

[111] Blanton LV Charbonneau MR Salih T Barratt MJ Venkatesh S Ilkaveya O. Gut bacteria that prevent growth impairments transmitted by microbiota from malnourished children. Science (2016) 351(6725). doi: 10.1126/science.aad3311 PMC4787260 26912898

[112] Stewart CJ Embleton ND Marrs EC Smith DP Nelson A Abdulkadir B. Temporal bacterial and metabolic development of the preterm gut reveals specific signatures in health and disease. Microbiome (2016) 4:67. doi: 10.1186/s40168-016-0216-8 28034304 PMC5200962

[113] Aagaard K Ma J Antony KM Ganu R Petrosino J Versalovic J. The placenta harbors a unique microbiome. Sci Transl Med (2014) 6:237ra265. doi: 10.1126/scitranslmed.3008599 PMC4929217 24848255

[114] Collado MC Rautava S Aakko J Isolauri E Salminen S. Human gut colonisation may be initiated *in utero* by distinct microbial communities in the placenta and amniotic fluid. Sci Rep (2016) 6:23129. doi: 10.1038/srep23129 27001291 PMC4802384

[115] Jimenez E Fernandez L Marin ML Martin R Odriozola JM Nueno-Palop C. Isolation of commensal bacteria from umbilical cord blood of healthy neonates born by cesarean section. Curr Microbiol (2005) 51:270–4. doi: 10.1007/s00284-005-0020-3 16187156

[116] Jimenez E Marin ML Martin R Odriozola JM Olivares M Xaus J. Is meconium from healthy newborns actually sterile? Res Microbiol (2008) 159:187–93. doi: 10.1016/j.resmic.2007.12.007 18281199

[117] Kimura I Miyamoto J Ohue-Kitano R Watanabe K Yamada T Onuki M. Maternal gut microbiota in pregnancy influences offspring metabolic phenotype in mice. Science (2020) 367(6481). doi: 10.1126/science.aaw8429 32108090

[118] Dominguez-Bello MG Costello EK Contreras M Magris M Hidalgo G Fierer N. Delivery mode shapes the acquisition and structure of the initial microbiota across multiple body habitats in newborns. Proc Natl Acad Sci USA (2010) 107:11971–5. doi: 10.1073/pnas.1002601107 PMC2900693 20566857

[119] Bäckhed F Roswall J Peng Y Feng Q Jia H Kovatcheva-Datchary P. Dynamics and stabilization of the human gut microbiome during the first year of life. Cell Host Microbe (2015) 17:690–703. doi: 10.1016/j.chom.2015.04.004 25974306

[120] Rutayisire E Huang K Liu Y Tao F. The mode of delivery affects the diversity and colonization pattern of the gut microbiota during the first year of infants' life: a systematic review. BMC Gastroenterol (2016) 16:86. doi: 10.1186/s12876-016-0498-0 27475754 PMC4967522

[121] Rotimi VO Duerden BI. The development of the bacterial flora in normal neonates. J Med Microbiol (1981) 14:51–62. doi: 10.1099/00222615-14-1-51 7463467

[122] Stark PL Lee A. The microbial ecology of the large bowel of breast-fed and formula-fed infants during the first year of life. J Med Microbiol (1982) 15:189–203. doi: 10.1099/00222615-15-2-189 7143428

[123] Yoshioka H Iseki K Fujita K. Development and differences of intestinal flora in the neonatal period in breast-fed and bottle-fed infants. Pediatrics (1983) 72:317–21. doi: 10.1542/peds.72.3.317 6412205

[124] Benno Y Sawada K Mitsuoka T. The intestinal microflora of infants: composition of fecal flora in breast-fed and bottle-fed infants. Microbiol Immunol (1984) 28:975–86. doi: 10.1111/j.1348-0421.1984.tb00754.x 6513816

[125] Adlerberth I Wold AE. Establishment of the gut microbiota in Western infants. Acta Paediatr (2009) 98:229–38. doi: 10.1111/j.1651-2227.2008.01060.x 19143664

[126] Palmer C Bik EM Digiulio DB Relman DA Brown PO. Development of the human infant intestinal microbiota. PloS Biol (2007) 5:e177. doi: 10.1371/journal.pbio.0050177 17594176 PMC1896187

[127] Roswall J Olsson LM Kovatcheva-Datchary P Nilsson S Tremaroli V Simon MC. Developmental trajectory of the healthy human gut microbiota during the first 5 years of life. Cell Host Microbe (2021) 29:765–776 e763. doi: 10.1016/j.chom.2021.02.021 33794185

[128] Jakobsson HE Abrahamsson TR Jenmalm MC Harris K Quince C Jernberg C. Decreased gut microbiota diversity, delayed Bacteroidetes colonisation and reduced Th1 responses in infants delivered by caesarean section. GUT (2014) 63(4):559–66. doi: 10.1136/gutjnl-2012-303249 23926244

[129] Penders J Thijs C Vink C Stelma FF Snijders B Kummeling I. Factors influencing the composition of the intestinal microbiota in early infancy. Pediatrics (2006) 118:511–21. doi: 10.1542/peds.2005-2824 16882802

[130] Erick M. Breast milk is conditionally perfect. Med Hypotheses (2018) 111:82–9. doi: 10.1016/j.mehy.2017.12.020 29407004

[131] Heikkilä MP Saris PEJ. Inhibition of staphylococcus aureus by the commensal bacteria of human milk. J Appl Microbiol (2003) 95:471–8. doi: 10.1046/j.1365-2672.2003.02002.x 12911694

[132] Martín R Langa S Reviriego C Jimínez E Marín ML Xaus J. Human milk is a source of lactic acid bacteria for the infant gut. J Pediatr (2003) 143:754–8. doi: 10.1016/j.jpeds.2003.09.028 14657823

[133] Beasley SS Saris PEJ. Nisin-producing lactococcus lactis strains isolated from human milk. Appl Environ Microbiol (2004) 70:5051–3. doi: 10.1128/AEM.70.8.5051-5053.2004 PMC492443 15294850

[134] Jiménez E Delgado S Maldonado A Arroyo R Albújar M García N. Staphylococcus epidermidis: a differential trait of the fecal microbiota of breast-fed infants. BMC Microbiol (2008) 8:143. doi: 10.1186/1471-2180-8-143 18783615 PMC2551609

[135] Jiménez E Fernández L Delgado S García N Albújar M Gómez A. Assessment of the bacterial diversity of human colostrum by cultural-based techniques. analysis of the staphylococcal and enterococcal populations. Res Microbiol (2008) 159:595–601. doi: 10.1016/j.resmic.2008.09.001 18845249

[136] Van Den Elsen LWJ Rekima A Verhasselt V. Early-life nutrition and gut immune development. Nestle Nutr Inst Workshop Ser (2019) 90:137–49. doi: 10.1159/000490301 30865982

[137] Koch MA Reiner GL Lugo KA Kreuk LS Stanbery AG Ansaldo E. Maternal IgG and IgA antibodies dampen mucosal T helper cell responses in early life. Cell (2016) 165:827–41. doi: 10.1016/j.cell.2016.04.055 PMC4866587 27153495

[138] Zheng W Zhao W Wu M Song X Caro F Sun X. Microbiota-targeted maternal antibodies protect neonates from enteric infection. Nature (2020) 577:543–8. doi: 10.1038/s41586-019-1898-4 PMC7362890 31915378

[139] Thurl S Munzert M Henker J Boehm G Muller-Werner B Jelinek J. Variation of human milk oligosaccharides in relation to milk groups and lactational periods. Br J Nutr (2010) 104:1261–71. doi: 10.1017/S0007114510002072 20522272

[140] Ballard O Morrow AL. Human milk composition: nutrients and bioactive factors. Pediatr Clin North Am (2013) 60:49–74. doi: 10.1016/j.pcl.2012.10.002 23178060 PMC3586783

[141] Locascio RG Desai P Sela DA Weimer B Mills DA. Broad conservation of milk utilization genes in bifidobacterium longum subsp. infantis as revealed by comparative genomic hybridization. Appl Environ Microbiol (2010) 76:7373–81. doi: 10.1128/AEM.00675-10 PMC2976205 20802066

[142] Koenig JE Spor A Scalfone N Fricker AD Stombaugh J Knight R. Succession of microbial consortia in the developing infant gut microbiome. Proc Natl Acad Sci USA (2011) 108:4578–85. doi: 10.1073/pnas.1000081107 PMC3063592 20668239

[143] Jost T Lacroix C Braegger CP Chassard C. New insights in gut microbiota establishment in healthy breast fed neonates. PloS One (2012) 7:e44595. doi: 10.1371/journal.pone.0044595 22957008 PMC3431319

[144] Henrick BM Yao XD Nasser L Roozrogousheh A Rosenthal KL. Breastfeeding behaviors and the innate immune system of human milk: working together to protect infants against inflammation, HIV-1, and other infections. Front Immunol (2017) 8:1631. doi: 10.3389/fimmu.2017.01631 29238342 PMC5712557

[145] Canfora EE Jocken JW Blaak EE. Short-chain fatty acids in control of body weight and insulin sensitivity. Nat Rev Endocrinol (2015) 11:577–91. doi: 10.1038/nrendo.2015.128 26260141

[146] Casaburi G Wei J Kazi S Liu J Wang K Tao GZ. Metabolic model of necrotizing enterocolitis in the premature newborn gut resulting from enteric dysbiosis. Front Pediatr (2022) 10:893059. doi: 10.3389/fped.2022.893059 36081629 PMC9445129

[147] Cabrera-Rubio R Collado MC Laitinen K Salminen S Isolauri E Mira A. The human milk microbiome changes over lactation and is shaped by maternal weight and mode of delivery. Am J Clin Nutr (2012) 96:544–51. doi: 10.3945/ajcn.112.037382 22836031

[148] Ward TL Hosid S Ioshikhes I Altosaar I. Human milk metagenome: a functional capacity analysis. BMC Microbiol (2013) 13:116. doi: 10.1186/1471-2180-13-116 23705844 PMC3679945

[149] Cabrera-Rubio R Mira-Pascual L Mira A Collado MC. Impact of mode of delivery on the milk microbiota composition of healthy women. J Dev Orig Health Dis (2016) 7:54–60. doi: 10.1017/S2040174415001397 26286040

[150] Aakko J Kumar H Rautava S Wise A Autran C Bode L. Human milk oligosaccharide categories define the microbiota composition in human colostrum. Benef Microbes (2017) 8:563–7. doi: 10.3920/BM2016.0185 28726512

[151] Cortes-Macías E Selma-Royo M García-Mantrana I Calatayud M González S Martínez-Costa C. Maternal diet shapes the breast milk microbiota composition and diversity: impact of mode of delivery and antibiotic exposure. J Nutr (2021) 151:330–40. doi: 10.1093/jn/nxaa310 PMC7850106 33188413

[152] El-Mohandes AE Keiser JF Johnson LA Refat M Jackson BJ. Aerobes isolated in fecal microflora of infants in the intensive care nursery: relationship to human milk use and systemic sepsis. Am J Infect Control (1993) 21:231–4. doi: 10.1016/0196-6553(93)90414-Y 8267233

[153] Hällström M Eerola E Vuento R Janas M Tammela O. Effects of mode of delivery and necrotising enterocolitis on the intestinal microflora in preterm infants. Eur J Clin Microbiol Infect Dis (2004) 23:463–70. doi: 10.1007/s10096-004-1146-0 15168141

[154] Verd S Porta R Botet F Gutiérrez A Ginovart G Barbero AH. Hospital outcomes of extremely low birth weight infants after introduction of donor milk to supplement mother's milk. Breastfeed Med (2015) 10:150–5. doi: 10.1089/bfm.2014.0138 25775218

[155] Hooper LV. Bacterial contributions to mammalian gut development. Trends Microbiol (2004) 12:129–34. doi: 10.1016/j.tim.2004.01.001 15001189

[156] Flint HJ Duncan SH Scott KP Louis P. Interactions and competition within the microbial community of the human colon: links between diet and health. Environ Microbiol (2007) 9:1101–11. doi: 10.1111/j.1462-2920.2007.01281.x 17472627

[157] Mazmanian SK Liu CH Tzianabos AO Kasper DL. An immunomodulatory molecule of symbiotic bacteria directs maturation of the host immune system. Cell (2005) 122:107–18. doi: 10.1016/j.cell.2005.05.007 16009137

[158] Martin R Nauta AJ Ben Amor K Knippels LM Knol J Garssen J. Early life: gut microbiota and immune development in infancy. Benef Microbes (2010) 1:367–82. doi: 10.3920/BM2010.0027 21831776

[159] Kaplan JL Shi HN Walker WA. The role of microbes in developmental immunologic programming. Pediatr Res (2011) 69:465–72. doi: 10.1203/PDR.0b013e318217638a 21364495

[160] Reikvam DH Erofeev A Sandvik A Grcic V Jahnsen FL Gaustad P. Depletion of murine intestinal microbiota: effects on gut mucosa and epithelial gene expression. PloS One (2011) 6:e17996. doi: 10.1371/journal.pone.0017996 21445311 PMC3061881

[161] El Aidy S Van Baarlen P Derrien M Lindenbergh-Kortleve DJ Hooiveld G Levenez F. Temporal and spatial interplay of microbiota and intestinal mucosa drive establishment of immune homeostasis in conventionalized mice. Mucosal Immunol (2012) 5:567–79. doi: 10.1038/mi.2012.32 22617837

[162] Hooper LV Littman DR Macpherson AJ. Interactions between the microbiota and the immune system. Science (2012) 336:1268–73. doi: 10.1126/science.1223490 PMC4420145 22674334

[163] Kang CS Ban M Choi EJ Moon HG Jeon JS Kim DK. Extracellular vesicles derived from gut microbiota, especially Akkermansia muciniphila, protect the progression of dextran sulfate sodium-induced colitis. PloS One (2013) 8:e76520. doi: 10.1371/journal.pone.0076520 24204633 PMC3811976

[164] Plovier H Everard A Druart C Depommier C Van Hul M Geurts L. A purified membrane protein from akkermansia muciniphila or the pasteurized bacterium improves metabolism in obese and diabetic mice. Nat Med (2017) 23:107–13. doi: 10.1038/nm.4236 27892954

[165] Martin R Chamignon C Mhedbi-Hajri N Chain F Derrien M Escribano-Vazquez U. The potential probiotic lactobacillus rhamnosus CNCM I-3690 strain protects the intestinal barrier by stimulating both mucus production and cytoprotective response. Sci Rep (2019) 9:5398. doi: 10.1038/s41598-019-41738-5 30931953 PMC6443702

[166] Ashrafian F Keshavarz Azizi Raftar S Shahryari A Behrouzi A Yaghoubfar R Lari A. Comparative effects of alive and pasteurized akkermansia muciniphila on normal diet-fed mice. Sci Rep (2021) 11:17898. doi: 10.1038/s41598-021-95738-5 34504116 PMC8429653

[167] Choi Y Bose S Seo J Shin JH Lee D Kim Y. Effects of live and pasteurized forms of akkermansia from the human gut on obesity and metabolic dysregulation. Microorganisms (2021) 9(10). doi: 10.3390/microorganisms9102039 PMC8538271 34683361

[168] Vaishnava S Behrendt CL Ismail AS Eckmann L Hooper LV. Paneth cells directly sense gut commensals and maintain homeostasis at the intestinal host-microbial interface. Proc Natl Acad Sci USA (2008) 105(52):20858–63. doi: 10.1073/pnas.0808723105 PMC2603261 19075245

[169] Rose EC Odle J Blikslager AT Ziegler AL. Probiotics, prebiotics and epithelial tight junctions: a promising approach to modulate intestinal barrier function. Int J Mol Sci (2021) 22(13). doi: 10.3390/ijms22136729 PMC8268081 34201613

[170] Marcobal A Barboza M Froehlich JW Block DE German JB Lebrilla CB. Consumption of human milk oligosaccharides by gut-related microbes. J Agric Food Chem (2010) 58:5334–40. doi: 10.1021/jf9044205 PMC2866150 20394371

[171] Marcobal A Sonnenburg JL. Human milk oligosaccharide consumption by intestinal microbiota. Clin Microbiol Infect (2012) 18 Suppl 4:12–5. doi: 10.1111/j.1469-0691.2012.03863.x PMC3671919 22647041

[172] Ninonuevo MR Park Y Yin H Zhang J Ward RE Clowers BH. A strategy for annotating the human milk glycome. J Agric Food Chem (2006) 54:7471–80. doi: 10.1021/jf0615810 17002410

[173] Niimi K Takahashi E. Reduced differentiation of intestinal epithelial cells in wasting marmoset syndrome. J Vet Med Sci (2021) 83:784–92. doi: 10.1292/jvms.20-0532 PMC8182325 33731497

[174] Bolick DT Chen T Alves LA Tong Y Wu D Joyner LT 2nd. Intestinal cell kinase is a novel participant in intestinal cell signaling responses to protein malnutrition. PloS One (2014) 9:e106902. doi: 10.1371/journal.pone.0106902 25184386 PMC4153720

[175] Yousefi M Nakauka-Ddamba A Berry CT Li N Schoenberger J Simeonov KP. Calorie restriction governs intestinal epithelial regeneration through cell-autonomous regulation of mTORC1 in reserve stem cells. Stem Cell Rep (2018) 10:703–11. doi: 10.1016/j.stemcr.2018.01.026 PMC5919411 29478893

[176] Moore SR Guedes MM Costa TB Vallance J Maier EA Betz KJ. Glutamine and alanyl-glutamine promote crypt expansion and mTOR signaling in murine enteroids. Am J Physiol Gastrointest Liver Physiol (2015) 308:G831–839. doi: 10.1152/ajpgi.00422.2014 PMC4437023 25792564

[177] Ueno PM Oria RB Maier EA Guedes M De Azevedo OG Wu D. Alanyl-glutamine promotes intestinal epithelial cell homeostasis *in vitro* and in a murine model of weanling undernutrition. Am J Physiol Gastrointest Liver Physiol (2011) 301:G612–622. doi: 10.1152/ajpgi.00531.2010 PMC3191556 21799183

[178] Qi M Tan B Wang J Liao S Li J Cui Z. Postnatal growth retardation is associated with deteriorated intestinal mucosal barrier function using a porcine model. J Cell Physiol (2021) 236:2631–48. doi: 10.1002/jcp.30028 32853405

[179] Takano J. Intestinal changes in protein-deficient rats. Exp Mol Pathol (1964) 3:224–31. doi: 10.1016/0014-4800(64)90055-3 14194322

[180] Hill RB Jr. Prosper J Hirschfield JS Kern F Jr. Protein starvation and the small intestine. i. the growth and morphology of the small intestine in weanling rats. Exp Mol Pathol (1968) 8:66–74. doi: 10.1016/0014-4800(68)90006-3 5637108

[181] Neutra MR Maner JH Mayoral LG. Effects of protein-calorie malnutrition on the jejunal mucosa of tetracycline-treated pigs. Am J Clin Nutr (1974) 27:287–95. doi: 10.1093/ajcn/27.3.287 4205494

[182] Nunez MC Bueno JD Ayudarte MV Almendros A Rios A Suarez MD. Dietary restriction induces biochemical and morphometric changes in the small intestine of nursing piglets. J Nutr (1996) 126:933–44. doi: 10.1093/jn/126.4.933 8613897

[183] Lopez-Pedrosa JM Torres MI Fernandez MI Rios A Gil A. Severe malnutrition alters lipid composition and fatty acid profile of small intestine in newborn piglets. J Nutr (1998) 128:224–33. doi: 10.1093/jn/128.2.224 9446848

[184] Van Der Sluis M De Koning BA De Bruijn AC Velcich A Meijerink JP Van Goudoever JB. Muc2-deficient mice spontaneously develop colitis, indicating that MUC2 is critical for colonic protection. Gastroenterology (2006) 131:117–29. doi: 10.1053/j.gastro.2006.04.020 16831596

[185] He W Wang ML Jiang HQ Steppan CM Shin ME Thurnheer MC. Bacterial colonization leads to the colonic secretion of RELMbeta/FIZZ2, a novel goblet cell-specific protein. Gastroenterology (2003) 125:1388–97. doi: 10.1016/j.gastro.2003.07.009 14598255

[186] Powell DN Swimm A Sonowal R Bretin A Gewirtz AT Jones RM. Indoles from the commensal microbiota act via the AHR and IL-10 to tune the cellular composition of the colonic epithelium during aging. Proc Natl Acad Sci USA (2020) 117(35):21519–26. doi: 10.1073/pnas.2003004117 PMC7474656 32817517

[187] Van Landeghem L Santoro MA Krebs AE Mah AT Dehmer JJ Gracz AD. Activation of two distinct Sox9-EGFP-expressing intestinal stem cell populations during crypt regeneration after irradiation. Am J Physiol Gastrointest Liver Physiol (2012) 302:G1111–1132. doi: 10.1152/ajpgi.00519.2011 PMC3362093 22361729

[188] Camilleri M Nadeau A Lamsam J Nord SL Ryks M Burton D. Understanding measurements of intestinal permeability in healthy humans with urine lactulose and mannitol excretion. Neurogastroenterol Motil (2010) 22(1):e15–26. doi: 10.1111/j.1365-2982.2009.01361.x PMC2802677 19614866

[189] Du J Chen Y Shi Y Liu T Cao Y Tang Y. 1,25-dihydroxyvitamin D protects intestinal epithelial barrier by regulating the myosin light chain kinase signaling pathway. Inflamm Bowel Dis (2015) 21:2495–506. doi: 10.1097/MIB.0000000000000526 PMC4646414 26287999

[190] Subramanian S Huq S Yatsunenko T Haque R Mahfuz M Alam MA. Persistent gut microbiota immaturity in malnourished Bangladeshi children. Nature (2014) 510:417–21. doi: 10.1038/nature13421 PMC4189846 24896187

[191] Iddrisu I Monteagudo-Mera A Poveda C Pyle S Shahzad M Andrews S. Malnutrition and gut microbiota in children. Nutrients (2021) 13(8). doi: 10.3390/nu13082727 PMC8401185 34444887

[192] Cowardin CA Ahern PP Kung VL Hibberd MC Cheng J Guruge JL. Mechanisms by which sialylated milk oligosaccharides impact bone biology in a gnotobiotic mouse model of infant undernutrition. Proc Natl Acad Sci USA (2019) 116(24):11988–96. doi: 10.1073/pnas.1821770116 PMC6575181 31138692

[193] Chen RY Mostafa I Hibberd MC Das S Mahfuz M Naila NN. A microbiota-directed food intervention for undernourished children. N Engl J Med (2021) 384:1517–28. doi: 10.1056/NEJMoa2023294 PMC7993600 33826814

[194] Erkelens MN Goverse G Konijn T Molenaar R Beijer MR Van Den Bossche J. Intestinal macrophages balance inflammatory expression profiles via vitamin A and dectin-1-mediated signaling. Front Immunol (2020) 11:551. doi: 10.3389/fimmu.2020.00551 32296441 PMC7138104

[195] Ochi T Feng Y Kitamoto S Nagao-Kitamoto H Kuffa P Atarashi K. Diet-dependent, microbiota-independent regulation of IL-10-producing lamina propria macrophages in the small intestine. Sci Rep (2016) 6:27634. doi: 10.1038/srep27634 27302484 PMC4908404

[196] Morhardt TL Hayashi A Kao JY Kamada N. Regional control of regulatory immune cells in the intestine. Curr Pathobiol Rep (2018) 6:29–34. doi: 10.1007/s40139-018-0156-z 29755892 PMC5943041

[197] Chng SH Kundu P Dominguez-Brauer C Teo WL Kawajiri K Fujii-Kuriyama Y. Ablating the aryl hydrocarbon receptor (AhR) in CD11c+ cells perturbs intestinal epithelium development and intestinal immunity. Sci Rep (2016) 6:23820. doi: 10.1038/srep23820 27068235 PMC4828637

[198] Xu X Dong Q Zhong Q Xiu W Chen Q Wang J. The flavonoid kurarinone regulates macrophage functions via aryl hydrocarbon receptor and alleviates intestinal inflammation in irritable bowel syndrome. J Inflamm Res (2021) 14:4347–59. doi: 10.2147/JIR.S329091 PMC8446718 34539182

[199] Iwata M Hirakiyama A Eshima Y Kagechika H Kato C Song SY. Retinoic acid imprints gut-homing specificity on T cells. Immunity (2004) 21:527–38. doi: 10.1016/j.immuni.2004.08.011 15485630

[200] Ooi JH Li Y Rogers CJ Cantorna MT. Vitamin D regulates the gut microbiome and protects mice from dextran sodium sulfate-induced colitis. J Nutr (2013) 143:1679–86. doi: 10.3945/jn.113.180794 PMC3771816 23966330

[201] Bruce D Yu S Ooi JH Cantorna MT. Converging pathways lead to overproduction of IL-17 in the absence of vitamin D signaling. Int Immunol (2011) 23:519–28. doi: 10.1093/intimm/dxr045 PMC3139478 21697289

[202] Ko HJ Hong SW Verma R Jung J Lee M Kim N. Dietary glucose consumption promotes RALDH activity in small intestinal CD103(+)CD11b(+) dendritic cells. Front Immunol (2020) 11:1897. doi: 10.3389/fimmu.2020.01897 32849649 PMC7433714

[203] Kudsk KA Carpenter G Petersen S Sheldon GF. Effect of enteral and parenteral feeding in malnourished rats with e. coli-hemoglobin adjuvant peritonitis. J Surg Res (1981) 31:105–10. doi: 10.1016/0022-4804(81)90037-8 6790873

[204] Kudsk KA Stone JM Carpenter G Sheldon GF. Enteral and parenteral feeding influences mortality after hemoglobin-e. coli peritonitis in normal rats. J Trauma (1983) 23:605–9. doi: 10.1097/00005373-198307000-00010 6410081

[205] Higashizono K Fukatsu K Watkins A Watanabe T Noguchi M Ri M. Influences of short-term fasting and carbohydrate supplementation on gut immunity and mucosal morphology in mice. JPEN J Parenter Enteral Nutr (2019) 43(4):516–24. doi: 10.1002/jpen.1446 30260489

[206] Reddy V Raghuramulu N Bhaskaram C. Secretory IgA in protein-calorie malnutrition. Arch Dis Child (1976) 51(11):871–4. doi: 10.1136/adc.51.11.871 PMC1546061 827242

[207] Mcclave SA Taylor BE Martindale RG Warren MM Johnson DR Braunschweig C. Guidelines for the provision and assessment of nutrition support therapy in the adult critically ill patient: society of critical care medicine (SCCM) and American society for parenteral and enteral nutrition (A.S.P.E.N.). JPEN J Parenter Enteral Nutr (2016) 40(2):159–211. doi: 10.1177/0148607115621863 26773077

[208] Pierre JF Busch RA Kudsk KA. The gastrointestinal immune system: implications for the surgical patient. Curr Probl Surg (2016) 53(1):11–47. doi: 10.1067/j.cpsurg.2015.10.005 26699624 PMC4811185

[209] Pérez-Romero M Serralde-Zúñiga AE Del Carmen Reyes-Ramírez AL Alfonso-Baruch E Gulias-Herrero A Castillo-Martínez L. Prevalence of malnutrition at admission in hospitalized adults at INCMNSZ in Mexico city. Rev Mex Endocrinol Metab Nutr (2017) 4(4).

[210] Kau AL Planer JD Liu J Rao S Yatsunenko T Trehan I. Functional characterization of IgA-targeted bacterial taxa from undernourished Malawian children that produce diet-dependent enteropathy. Sci Transl Med (2015) 7:276ra224. doi: 10.1126/scitranslmed.aaa4877 PMC4423598 25717097

[211] Huus KE Bauer KC Brown EM Bozorgmehr T Woodward SE Serapio-Palacios A. Commensal bacteria modulate immunoglobulin A binding in response to host nutrition. Cell Host Microbe (2020) 27:909–921 e905. doi: 10.1016/j.chom.2020.03.012 32289261

[212] Sun J Qi C Zhu H Zhou Q Xiao H Le G. IgA-targeted lactobacillus jensenii modulated gut barrier and microbiota in high-fat diet-fed mice. Front Microbiol (2019) 10:1179. doi: 10.3389/fmicb.2019.01179 31178854 PMC6542990

[213] Bourke CD Berkley JA Prendergast AJ. Immune dysfunction as a cause and consequence of malnutrition. Trends Immunol (2016) 37:386–98. doi: 10.1016/j.it.2016.04.003 PMC4889773 27237815

[214] Albenberg LG Wu GD. Diet and the intestinal microbiome: associations, functions, and implications for health and disease. Gastroenterology (2014) 146:1564–72. doi: 10.1053/j.gastro.2014.01.058 PMC4216184 24503132

[215] Ghosh TS Gupta SS Bhattacharya T Yadav D Barik A Chowdhury A. Gut microbiomes of Indian children of varying nutritional status. PloS One (2014) 9:e95547. doi: 10.1371/journal.pone.0095547 24763225 PMC3999041

[216] Gentile CL Weir TL. The gut microbiota at the intersection of diet and human health. Science (2018) 362:776–80. doi: 10.1126/science.aau5812 PMC13264711 30442802

[217] Wu Y Wan J Choe U Pham Q Schoene NW He Q. Interactions between food and gut microbiota: impact on human health. Annu Rev Food Sci Technol (2019) 10:389–408. doi: 10.1146/annurev-food-032818-121303 30908952

[218] Ecklu-Mensah G Gilbert J Devkota S. Dietary selection pressures and their impact on the gut microbiome. Cell Mol Gastroenterol Hepatol (2022) 13:7–18. doi: 10.1016/j.jcmgh.2021.07.009 34329765 PMC8600059

[219] Monira S Nakamura S Gotoh K Izutsu K Watanabe H Alam NH. Gut microbiota of healthy and malnourished children in bangladesh. Front Microbiol (2011) 2:228. doi: 10.3389/fmicb.2011.00228 22125551 PMC3221396

[220] Hashimoto T Perlot T Rehman A Trichereau J Ishiguro H Paolino M. ACE2 links amino acid malnutrition to microbial ecology and intestinal inflammation. Nature (2012) 487:477–81. doi: 10.1038/nature11228 PMC7095315 22837003

[221] Smith MI Yatsunenko T Manary MJ Trehan I Mkakosya R Cheng J. Gut microbiomes of Malawian twin pairs discordant for kwashiorkor. Science (2013) 339:548–54. doi: 10.1126/science.1229000 PMC3667500 23363771

[222] Preidis GA Ajami NJ Wong MC Bessard BC Conner ME Petrosino JF. Composition and function of the undernourished neonatal mouse intestinal microbiome. J Nutr Biochem (2015) 26:1050–7. doi: 10.1016/j.jnutbio.2015.04.010 26070414

[223] Ghosh S Whitley CS Haribabu B Jala VR. Regulation of intestinal barrier function by microbial metabolites. Cell Mol Gastroenterol Hepatol (2021) 11:1463–82. doi: 10.1016/j.jcmgh.2021.02.007 PMC8025057 33610769

[224] Million M Tidjani Alou M Khelaifia S Bachar D Lagier JC Dione N. Increased gut redox and depletion of anaerobic and methanogenic prokaryotes in severe acute malnutrition. Sci Rep (2016) 6:26051. doi: 10.1038/srep26051 27183876 PMC4869025

[225] Gehrig JL Venkatesh S Chang HW Hibberd MC Kung VL Cheng J. Effects of microbiota-directed foods in gnotobiotic animals and undernourished children. Science (2019) 365(6449). doi: 10.1126/science.aau4732 PMC6683325 31296738

[226] Raman AS Gehrig JL Venkatesh S Chang HW Hibberd MC Subramanian S. A sparse covarying unit that describes healthy and impaired human gut microbiota development. Science (2019) 365(6449). doi: 10.1126/science.aau4735 PMC6683326 31296739

[227] Popkin BM Corvalan C Grummer-Strawn LM. Dynamics of the double burden of malnutrition and the changing nutrition reality. Lancet (2020) 395:65–74. doi: 10.1016/S0140-6736(19)32497-3 31852602 PMC7179702

[228] Guiraldes E Hamilton JR. Effect of chronic malnutrition on intestinal structure, epithelial renewal, and enzymes in suckling rats. Pediatr Res (1981) 15:930–4. doi: 10.1203/00006450-198106000-00010 6787548

[229] Schwarzer M Makki K Storelli G Machuca-Gayet I Srutkova D Hermanova P. Lactobacillus plantarum strain maintains growth of infant mice during chronic undernutrition. Science (2016) 351:854–7. doi: 10.1126/science.aad8588 26912894

[230] Jones EA Waldmann TA. The mechanism of intestinal uptake and transcellular transport of IgG in the neonatal rat. J Clin Invest (1972) 51:2916–27. doi: 10.1172/JCI107116 PMC292442 5080417

[231] Lykke M Hother AL Hansen CF Friis H Molgaard C Michaelsen KF. Malnutrition induces gut atrophy and increases hepatic fat infiltration: studies in a pig model of childhood malnutrition. Am J Transl Res (2013) 5(5):543–54. PMC3745441 23977413

[232] Clarke RM Hardy RN. Structural changes in the small intestine associated with the uptake of polyvinyl pyrrolidone by the young ferret, rabbit, guinea-pig, cat and chicken. J Physiol (1970) 209:669–87. doi: 10.1113/jphysiol.1970.sp009185 PMC1395547 5499802

[233] Westrom BR Svendsen J Ohlsson BG Tagesson C Karlsson BW. Intestinal transmission of macromolecules (BSA and FITC-labelled dextrans) in the neonatal pig. influence of age of piglet and molecular weight of markers. Biol Neonate (1984) 46:20–6. doi: 10.1159/000242028 6204696

[234] Jakobsson I Lindberg T Lothe L Axelsson I Benediktsson B. Human alpha-lactalbumin as a marker of macromolecular absorption. Gut (1986) 27:1029–34. doi: 10.1136/gut.27.9.1029 PMC1433798 3758816

[235] Rodriguez P Darmon N Chappuis P Candalh C Blaton MA Bouchaud C. Intestinal paracellular permeability during malnutrition in guinea pigs: effect of high dietary zinc. Gut (1996) 39:416–22. doi: 10.1136/gut.39.3.416 PMC1383349 8949647

[236] Hamada H Hiroi T Nishiyama Y Takahashi H Masunaga Y Hachimura S. Identification of multiple isolated lymphoid follicles on the antimesenteric wall of the mouse small intestine. J Immunol (2002) 168:57–64. doi: 10.4049/jimmunol.168.1.57 11751946

[237] Russell GJ Bhan AK Winter HS. The distribution of T and b lymphocyte populations and MHC class II expression in human fetal and postnatal intestine. Pediatr Res (1990) 27:239–44. doi: 10.1203/00006450-199003000-00007 2320390

[238] Spencer J Klavinskis LS Fraser LD. The human intestinal IgA response; burning questions. Front Immunol (2012) 3:108. doi: 10.3389/fimmu.2012.00108 22593756 PMC3349913

[239] Gustafson CE Higbee D Yeckes AR Wilson CC De Zoeten EF Jedlicka P. Limited expression of APRIL and its receptors prior to intestinal IgA plasma cell development during human infancy. Mucosal Immunol (2014) 7:467–77. doi: 10.1038/mi.2013.64 PMC3959635 24045575

[240] Hill DR Spence JR. Gastrointestinal organoids: understanding the molecular basis of the host-microbe interface. Cell Mol Gastroenterol Hepatol (2017) 3(2):138–49. doi: 10.1016/j.jcmgh.2016.11.007 PMC5331777 28275681

[241] Afrazi A Branca MF Sodhi CP Good M Yamaguchi Y Egan CE. Toll-like receptor 4-mediated endoplasmic reticulum stress in intestinal crypts induces necrotizing enterocolitis. J Biol Chem (2014) 289(14):9584–99. doi: 10.1074/jbc.M113.526517 PMC3975009 24519940

[242] Sodhi CP Wipf P Yamaguchi Y Fulton WB Kovler M Nino DF. The human milk oligosaccharides 2'-fucosyllactose and 6'-sialyllactose protect against the development of necrotizing enterocolitis by inhibiting toll-like receptor 4 signaling. Pediatr Res (2021) 89(1):91–101. doi: 10.1038/s41390-020-0852-3 32221473 PMC7529714

[243] Kovbasnjuk O Zachos NC In J Foulke-Abel J Ettayebi K Hyser JM. Human enteroids: preclinical models of non-inflammatory diarrhea. Stem Cell Res Ther (2013) 4 Suppl 1:S3. doi: 10.1186/scrt364 24564938 PMC4029787

[244] Foulke-Abel J In J Kovbasnjuk O Zachos NC Ettayebi K Blutt SE. Human enteroids as an *ex-vivo* model of host-pathogen interactions in the gastrointestinal tract. Exp Biol Med (Maywood) (2014) 239(9):1124–34. doi: 10.1177/1535370214529398 PMC4380516 24719375

[245] Drummond CG Bolock AM Ma C Luke CJ Good M Coyne CB. Enteroviruses infect human enteroids and induce antiviral signaling in a cell lineage-specific manner. Proc Natl Acad Sci USA (2017) 114(7):1672–7. doi: 10.1073/pnas.1617363114 PMC5320971 28137842

[246] Lanik WE Mara MA Mihi B Coyne CB Good M. Stem cell-derived models of viral infections in the gastrointestinal tract. Viruses (2018) 10(3). doi: 10.3390/v10030124 PMC5869517 29534451

[247] Engevik MA Danhof HA Chang-Graham AL Spinler JK Engevik KA Herrmann B. Human intestinal enteroids as a model of clostridioides difficile-induced enteritis. Am J Physiol Gastrointest Liver Physiol (2020) 318:G870–88. doi: 10.1152/ajpgi.00045.2020 PMC7272722 32223302

[248] Ruan W Engevik MA Chang-Graham AL Danhof HA Goodwin A Engevik KA. Enhancing responsiveness of human jejunal enteroids to host and microbial stimuli. J Physiol (2020) 598:3085–105. doi: 10.1113/JP279423 PMC7674265 32428244

[249] Ingle H Hassan E Gawron J Mihi B Li Y Kennedy EA. Murine astrovirus tropism for goblet cells and enterocytes facilitates an IFN-lambda response *in vivo* and in enteroid cultures. Mucosal Immunol (2021) 14:751–61. doi: 10.1038/s41385-021-00387-6 PMC8085034 33674763

[250] Gracz AD Williamson IA Roche KC Johnston MJ Wang F Wang Y. A high-throughput platform for stem cell niche co-cultures and downstream gene expression analysis. Nat Cell Biol (2015) 17:340–9. doi: 10.1038/ncb3104 PMC4405128 25664616

[251] Samsa LA Williamson IA Magness ST. Quantitative analysis of intestinal stem cell dynamics using microfabricated cell culture arrays. Methods Mol Biol (2018) 1842:139–66. doi: 10.1007/978-1-4939-8697-2_10 30196407

[252] Iqbal NT Syed S Sadiq K Khan MN Iqbal J Ma JZ. Study of environmental enteropathy and malnutrition (SEEM) in Pakistan: protocols for biopsy based biomarker discovery and validation. BMC Pediatr (2019) 19:247. doi: 10.1186/s12887-019-1564-x 31331393 PMC6643315

